# Mechanistic evaluation of NSC 57774 as a SHP2 inhibitor in gastric cancer: Multi-pathway signaling modulation in vitro

**DOI:** 10.1371/journal.pone.0354605

**Published:** 2026-07-30

**Authors:** Ghalia Khoder, Rose Ghemrawi, Nour Sammani, Rania Harati, Mohamad Hamad, Jibran Sualeh Muhammad, Walaa Mousa, Mostafa Khair

**Affiliations:** 1 Department of Pharmaceutics and Pharmaceuticals Technology, College of Pharmacy, University of Sharjah, United Arab Emirates; 2 Research Institute for Medical & Health Sciences, University of Sharjah, United Arab Emirates; 3 College of Pharmacy, Al Ain University, Abu Dhabi, United Arab Emirates; 4 AAU Health and Biomedical Research Center, Al Ain University, Abu Dhabi, United Arab Emirates; 5 Department of Pharmacy Practice and Pharmacotherapeutics, College of Pharmacy, University of Sharjah, Sharjah, United Arab Emirates; 6 Department of Medical Laboratory Sciences, College of Health Sciences, University of Sharjah, United Arab Emirates; 7 Department of Biomedical Sciences, College of Medicine and Health, University of Birmingham, Birmingham, United Kingdom; 8 Core Technology Platforms, New York University Abu Dhabi, Abu Dhabi, United Arab Emirates; China Medical University, TAIWAN

## Abstract

Gastric cancer (GC) remains a leading cause of cancer-related mortality worldwide, driven by late-stage diagnosis, metastatic progression, and therapeutic resistance. Src homology region 2 domain-containing phosphatase 2 (SHP2) has emerged as a critical regulator of oncogenic signaling in gastric tumorigenesis, yet its therapeutic targeting remains underexplored. In this study, we evaluated the anti-cancer efficacy of NSC 57774, a novel SHP2 inhibitor, using integrated bioinformatics and functional assays in AGS gastric cancer cells. Analysis of The Cancer Genome Atlas (TCGA) and UALCAN datasets revealed marked upregulation of SHP2 and multiple receptor tyrosine kinases in gastric cancer tissues. NSC 57774 potently inhibited cell proliferation and migration, demonstrating selective cytotoxicity towards cancer cells over non-cancerous fibroblasts. Mechanistically, NSC 57774 disrupted key oncogenic pathways including MAPK/ERK, AKT and STAT3 in a concentration- and time-dependent manner, with higher doses achieving more sustained pathway suppression. NSC 57774 suppressed NF-κB inflammatory signaling at early timepoints and induced cleaved caspase-3 across all treatment groups at 72 hours, indicative of pro-apoptotic activity. A paradoxical late-phase increase in phospho-p38 was observed at 72 hours, consistent with a compensatory pro-apoptotic stress response. Comparative analysis revealed that NSC 57774 outperformed the commercial SHP2 inhibitor NSC 87877 and doxorubicin in reducing viability and migration of gastric cancer cells. Collectively, these findings position NSC 57774 as a promising candidate for targeted gastric cancer therapy, capable of disrupting multiple signaling pathways involved in tumor progression, metastasis, and inflammation, warranting further preclinical and clinical investigation.

## Introduction

Gastric cancer (GC) ranks as the fifth most common malignancy worldwide and is the fourth leading cause of cancer-related death [1]. The 5-year survival rate for this disease remains distressingly low, ranging from 20% to 40% across different countries, largely due to late-stage diagnosis and resistance to initial treatments [2]. Among the potential targets for therapy, Receptor Tyrosine Kinases (RTKs) are frequently found to be abnormally activated in various cancers, including GC, driving tumor growth and progression [3]. A key player in this process is the non-receptor protein tyrosine phosphatase SHP2, encoded by the PTPN11 gene. Mutations in PTPN11 are linked to increased tumor development due to conditions like Noonan syndrome and Leopard syndrome [4] and SHP2 mutations are recurrently observed in various cancers [5]. SHP2’s role extends to mediating signals from multiple RTKs and regulating essential cellular processes such as growth, differentiation, migration and apoptosis, particularly through the Ras/MAPK pathway [6,7]. In the context of GC, SHP2 not only promotes tumor growth but also represents a promising therapeutic target. Its activity becomes particularly elevated following the amplification of RTKs, such as Met and fibroblast growth factor receptor 2 (FGFR2) [8]. Strategies to diminish SHP2 function, through genetic knockdown or pharmacological inhibition, have shown promise in curbing the proliferation of cancer cells and their metastatic spread [8]. Furthermore, SHP2 is implicated in the progression of gastric adenocarcinoma by transmitting oncogenic signals essential for MAPK pathway activation, which are crucial for cell proliferation and survival [9]. Notably, SHP2 is also linked to gastric adenocarcinoma development related to *Helicobacter pylori (H. pylori)* infection. Among the oncogenic mechanisms driving SHP2 hyperactivation in gastric cancer, the interaction between SHP2 and the *H. pylori* virulence protein CagA is particularly well-characterized. Upon translocation into gastric epithelial cells, CagA directly binds and activates SHP2 at the post-translational level, promoting oncogenic signaling independently of PTPN11 transcriptional upregulation [10–13].

To date, limited studies have explored the effects of SHP2 inhibition in various types of gastric cancer and there is insufficient data regarding the outcomes of SHP2 inhibition in gastric cancer, whether associated with *H. pylori* infection or not. This study critically evaluates the SHP2 inhibitor NSC 57774, previously identified for its effectiveness in breast cancer [14], to assess its therapeutic potential in gastric cancer. By extending our investigation into gastric cancer, we aim to determine the efficacy of NSC 57774 in inhibiting tumor growth and disrupting survival and inflammation pathways. Our findings demonstrate that NSC 57774 significantly reduces the proliferation of AGS gastric cancer cells, inhibits their migration and suppresses inflammation by targeting the MAPK/ERK, JAK/STAT and FAK/ NF-κB/P38 pathways. This study enriches our understanding of SHP2’s role in gastric cancer and paves the way for the development of targeted therapies that address tumor proliferation, metastasis and inflammation. It also lays the foundation for future investigations into the crosstalk between the *H. pylori* CagA oncoprotein and SHP2-mediated signaling pathways.

## Materials and methods

### UALCAN analysis

Gene expression analyses were conducted using the UALCAN web platform (http://ualcan.path.uab.edu), which integrates high-throughput data from The Cancer Genome Atlas (TCGA) and the Clinical Proteomic Tumor Analysis Consortium (CPTAC) [15]. This tool was employed to evaluate the expression profiles of the PTPN11 gene, encoding SHP2, as well as RTKs in gastric tissue samples. Comparisons were made between stomach adenocarcinoma (STAD) (n = 415) and adjacent normal tissues (n = 34), including subsets stratified by *H. pylori* infection status, to identify differential expression patterns relevant to disease progression and microbial influence. Gene expression analyses via UALCAN were performed using the platform’s built-in Wilcoxon rank-sum test for comparisons between tumor and normal groups. Results were considered significant at P < 0.05, as reported by the platform. As UALCAN does not provide raw expression values, Table 1 reports direction of change (upregulated/downregulated/insignificant) based on platform-reported significance thresholds.

**Table 1:** SHP2 and receptor tyrosine kinases (RTK) expression levels using the stomach adenocarcinoma (STAD) with and without *H. pylori* infection from TCGA using UALCAN platform (http://ualcan.path.uab.edu/cgi-bin/).

Genes	Gene expression in primary STAD	Gene expression in STAD (without HP infection)-vs-STAD (with HP infection)	Gene expression in STAD vs Normal (without HP infection)	Gene expression in STAD vs Normal (with HP infection)
**SHP2 encoding gene**				
**PTPN11**	Upregulated*	Insignificant change	Upregulated*	Upregulated*
**Receptor Tyrosine Kinase genes**				
**EGFR**	Upregulated*	Downregulated*	Insignificant change	Insignificant change
**ERBB2**	Upregulated*	Insignificant change	Upregulated*	Insignificant change
**ERBB3**	Upregulated*	Downregulated*	Upregulated*	Upregulated*
**INSR**	Upregulated*	Insignificant change	Upregulated*	Upregulated*
**FGFR2**	Upregulated*	Insignificant change	Upregulated*	Insignificant change
**FGFR4**	Upregulated*	Insignificant change	Upregulated*	Upregulated*
**PDGFRB**	Upregulated*	Insignificant change	Upregulated*	Upregulated*
**FLT1**	Upregulated*	Insignificant change	Upregulated*	Upregulated*
**KDR**	Upregulated*	Insignificant change	Upregulated*	Upregulated*
**FLT4**	Upregulated*	Insignificant change	Upregulated*	Upregulated*
**FGFRL1**	Upregulated*	Insignificant change	Upregulated*	Upregulated*
**EPHA2**	Upregulated*	Insignificant change	Upregulated*	Insignificant change
**EPHA10**	Upregulated*	Insignificant change	Upregulated*	Upregulated*
**EPHB1**	Upregulated*	Insignificant change	Upregulated*	Upregulated*
**EPHB2**	Upregulated*	Insignificant change	Upregulated*	Upregulated*
**DDR1**	Upregulated*	Insignificant change	Upregulated*	Upregulated*
**MET**	Upregulated*	Insignificant change	Upregulated*	Upregulated*
**MST1R**	Upregulated*	Downregulated*	Upregulated*	Upregulated*
**ROS1**	Upregulated*	Insignificant change	Upregulated*	Insignificant change
**TIE1**	Upregulated*	Insignificant change	Upregulated*	Upregulated*
**EPHA6**	Downregulated*	Insignificant change	Downregulated*	Downregulated*
**EPHA7**	Downregulated*	Insignificant change	Downregulated*	Downregulated*
**EPHA8**	Downregulated*	Upregulated*	Downregulated*	Upregulated*
**EPHB3**	Downregulated*	Insignificant change	Downregulated*	Insignificant change
**NTRK3**	Downregulated*	Insignificant change	Downregulated*	Downregulated*
**ALK**	Downregulated*	Insignificant change	Downregulated*	Downregulated*
**ERBB4**	Insignificant change	Downregulated*	Insignificant change	Downregulated*
**IGF1R**	Insignificant change	Insignificant change	Upregulated*	Insignificant change
**INSRR**	Insignificant change	Insignificant change	Insignificant change	Downregulated*
**FGFR1**	Insignificant change	Insignificant change	Downregulated*	Insignificant change
**FGFR3**	Insignificant change	Insignificant change	Insignificant change	Insignificant change
**PDGFRA**	Insignificant change	Insignificant change	Insignificant change	Insignificant change
**EPHA1**	Insignificant change	Downregulated*	Insignificant change	Insignificant change
**EPHA3**	Insignificant change	Insignificant change	Insignificant change	Insignificant change
**EPHA4**	Insignificant change	Insignificant change	Insignificant change	Downregulated*
**EPHA5**	Insignificant change	Insignificant change	Insignificant change	Insignificant change
**EPHB6**	Insignificant change	Insignificant change	Downregulated*	Insignificant change
**AXL**	Insignificant change	Insignificant change	Downregulated*	Insignificant change
**MERTK**	Insignificant change	Insignificant change	Insignificant change	Insignificant change
**TYRO3**	Insignificant change	Downregulated*	Insignificant change	Insignificant change
**DDR2**	Insignificant change	Insignificant change	Downregulated*	Insignificant change
**TEK**	Insignificant change	Insignificant change	Insignificant change	Insignificant change
**RET**	Insignificant change	Insignificant change	Insignificant change	Insignificant change
**NTRK1**	Insignificant change	Insignificant change	Insignificant change	Insignificant change
**NTRK2**	Insignificant change	Insignificant change	Insignificant change	Downregulated*
**RYK**	Insignificant change	Insignificant change	Insignificant change	Insignificant change
**LTK**	Insignificant change	Insignificant change	Insignificant change	Insignificant change

### SHP2 inhibitors

**Fig 1:**
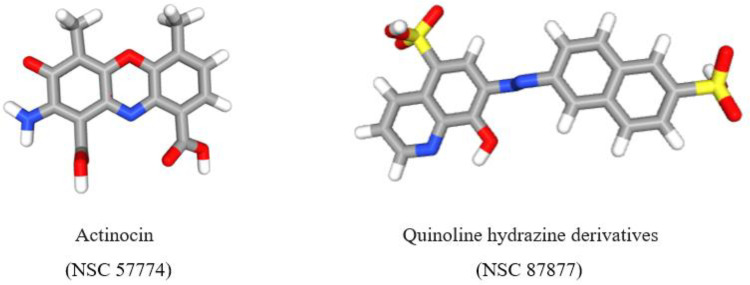
Chemical structures of SHP2 inhibitors (NSC 57774 and NSC 87877).

SHP2 inhibitors, including NSC 57774 (CAS: 524-11-8; Glixx Laboratories Inc.) and NSC 87877 (HY-18756; MedChem Express), are essential tools for targeting SHP2 in cancer research. NSC 87877, a widely used positive control, was selected due to its commercial availability and well-documented effectiveness in inhibiting SHP2 activity [16,17]. The chemical structures of the used SHP2 inhibitors were illustrated in Fig 1. The synthesis of NSC 57774 and its derivatives was previously described [14] where it demonstrated efficacy as an SHP2 inhibitor in breast cancer models. Stock solutions of both NSC 57774 and NSC 87877 were prepared in 100% DMSO, with working solutions ensuring that DMSO concentrations did not exceed 1% to minimize solvent-related effects. The chemotherapeutic agent Doxorubicin Hydrochloride Salt (Dox) (# D4000, LC Laboratories) was used as an anticancer standard to validate the experimental results [18]. Notably, doxorubicin was included solely as a standard cytotoxic reference agent to benchmark the general anti-proliferative and anti-migratory potency of NSC 57774, rather than as a mechanistic comparator.

### Human cell culture

The Human gastric adenocarcinoma AGS cells (ATCC CRL-1739) were purchased from the German Cell Lines Service (CLS, Germany) and cultured in RPMI-1640 medium (#R8758, Sigma, USA) supplemented with 10% fetal bovine serum ((#F9665, FBS, Sigma, USA) and streptomycin-penicillin (100 µg/mL and 100 IU/mL) (#A5955, Sigma, USA). Cells were incubated at 37˚C in a humidified atmosphere with 5% CO2 until 80−90% confluency. Sub-culturing was conducted every 2−4 days to sustain cell viability. All experiments were conducted using cells in the exponential growth phase. Human dermal fibroblasts (HDF, ATCC PCS-201–012) cell line was procured from American Type Culture Collection (Manassas, VA, USA). The cells were cultured in DMEM supplemented with 10% (w/v) fetal calf serum, penicillin/streptomycin (100 U/mL). The culture conditions were maintained at 37°C under a 5% CO_2_ atmosphere and 95% relative humidity. Upon reaching confluence, the fibroblasts were detached from the culture flasks utilizing a 0.1% trypsin and 0.02% EDTA solution, subsequently washed and resuspended in the supplemented DMEM media. For subsequent experiments, HDF cells at passages 6–7 were utilized.

### Cell proliferation assay

The effect of SHP2 inhibitor (NSC 57774) on cellular viability was assessed via the MTT (3-(4, 5-dimethylthiazolyl-2)-2, 5-diphenyltetrazolium bromide) assay. AGS cells were seeded in 96-well plates at a density of 5 x 10^3^ cells/well and subsequently incubated at 37 °C and 5% CO_2_ for 24 h. AGS cells were exposed to varying concentrations of SHP2 inhibitor (NSC 57774), control SHP2 inhibitor (NSC 87877) and Doxorubicin (Dox) for 24, 48 and 72 hours.At each designated time point (24, 48 or 72 hours), 100 ul of MTT solution (50 µg/mL) was added to each well and the cells were incubated for additional 2h at 37˚C. The solubilization of MTT crystals was accomplished by adding 100 µl of DMSO followed by 10 min of incubation. The plates were shaken for 5 min to dissolve the MTT formazan crystals. The OD value of each well was determined using a Synergy H1 microplate reader (Biotek, USA) at a wavelength of 570 nm. The IC50 values were calculated using GraphPad Prism 9 (GraphPad Software Inc., La Jolla, CA, USA) by fitting normalized cell viability data (expressed as percentage of untreated control) to a four-parameter logistic non-linear regression model. The top and bottom constraints were set to 100% and 0% respectively, and the Hillslope was left unconstrained. R² values for all fitted curves exceeded 0.80, confirming adequate model fit, as reported in S1 Fig and S2 Fig. Each IC50 determination was derived from two independent experiments performed in triplicate.

### Wound healing assay

This assay is used to evaluate cell migration and proliferation. To this purpose, AGS cells were plated in 96-well plates and allowed to reach 70–80% confluence overnight. A uniform scratch was introduced across the cell monolayer using a sterile 200 µL pipette tip, followed by two washes with phosphate-buffered saline (PBS) to remove detached cell debris. Cells were then treated with NSC 57774, NSC 87877, Doxorubicin or vehicle control (DMSO) in serum-reduced medium (2% FBS in RPMI-1640) to minimise the confounding effect of serum-induced proliferation on wound closure measurements. Phase-contrast images were captured at 0, 24, 48 and 72 hours post-treatment and wound closure was quantified using the wound healing size tool in ImageJ software (National Institutes of Health, Bethesda, MD, USA). All assays were performed in four independent biological experiments, with a minimum of three fields of view analysed per scratch per time point.

### Protein extraction and western blot

Post-treatment with SHP2 inhibitors (NSC 5p-ERK774, NSC 87877) or DMSO for 24, 48, or 72 hours, AGS cells were lysed in RIPA buffer containing protease and phosphatase inhibitors. Lysates were centrifuged at 12,000 rpm for 20 minutes and the supernatant was used for protein quantification via the BCA Protein Assay (23225, Thermo Fisher Scientific, Waltham, MA, USA). For Western blotting, 20 µg of protein from each sample was loaded into SDS-PAGE, transferred to nitrocellulose membranes Membranes were blocked for 1 hour at room temperature with either 5% non-fat skimmed milk in Tween-TBS for detection of non-phosphorylated proteins or with 5% bovine serum albumin (BSA) in Tween-TBS for detection of phosphorylated proteins as BSA is required to prevent background interference from endogenous phosphoproteins present in milk. The membranes were then exposed to primary antibodies (1:700 dilution) overnight, targeting NF-κB p65 (D14E12) (#8242, Cell signaling, USA), Phospho-p44/42 MAPK (Erk1/2) (#4370, Cell signaling, USA), p44/42 MAPK (Erk1/2) (#4695, Cell signaling, USA), Phospho-Stat3 (Tyr705) (#9145, Cell signaling, USA), Stat3 (79D7) (#4904, Cell signaling, USA), Caspase-3 (#9662, Cell signaling, USA), Phospho-Akt (Ser473) (#9271, Cell signaling, USA), Akt (#9272, Cell signaling, USA), SHP-2 (#3752, Cell signaling, USA), Phospho-p38 MAPK (Thr180/Tyr182) (#9145, Cell signaling, USA), p38 MAPK (#9212, Cell signaling, USA), FAK (#3285, Cell signaling, USA) and GAPDH (#2118S, Cell signaling, USA). Following three washes in Tween-TBS (10 mM Tris- HCl, pH 7.5; 100 mM NaCl; 0.1% Tween-20), the membranes were then incubated with a secondary antibody (7074, Cell signaling, USA) (1:2000) for 1 hour. Detection was accomplished using appropriate secondary antibodies conjugated to HRP. Band densitometry was performed using Image Lab software (Bio-Rad, USA) using the Gel Analysis tool. For each blot, a region of interest was drawn consistently around each band and local background subtraction was applied to correct for non-specific signal. Band intensities of target proteins were normalised to the corresponding GAPDH loading control band from the same membrane. For phosphorylated proteins, the ratio of phosphorylated to total protein was calculated. All normalised values were subsequently expressed relative to the DMSO vehicle control group, which was set to 1.0, to allow direct comparison across treatment conditions. All Western blot experiments were performed in two independent biological replicates, and band intensities were quantified twice using Image Lab software. Results are expressed as mean ± SD.

### Statistical analysis

Data analysis was conducted using GraphPad Prism version 9 (GraphPad Software Inc., La Jolla, CA, USA). Means and standard deviations (SD) were calculated from two independent biological experiments performed on separate occasions. For comparisons between each treatment group and the DMSO vehicle control, Student’s unpaired t-test was applied. For inter-group comparisons across all treatment conditions, One-Way ANOVA with Dunnett’s multiple comparisons test was used. Results were considered statistically significant at P < 0.05 (*P < 0.05, **P < 0.01, ***P < 0.001). IC50 values were determined by non-linear regression using a four-parameter logistic curve fitting model, with top and bottom constraints set to 100% and 0% respectively, derived from two independent experiments each performed in triplicate.

## Results

### SHP2 and receptor tyrosine kinases expression in gastric cancer

**Fig 2:**
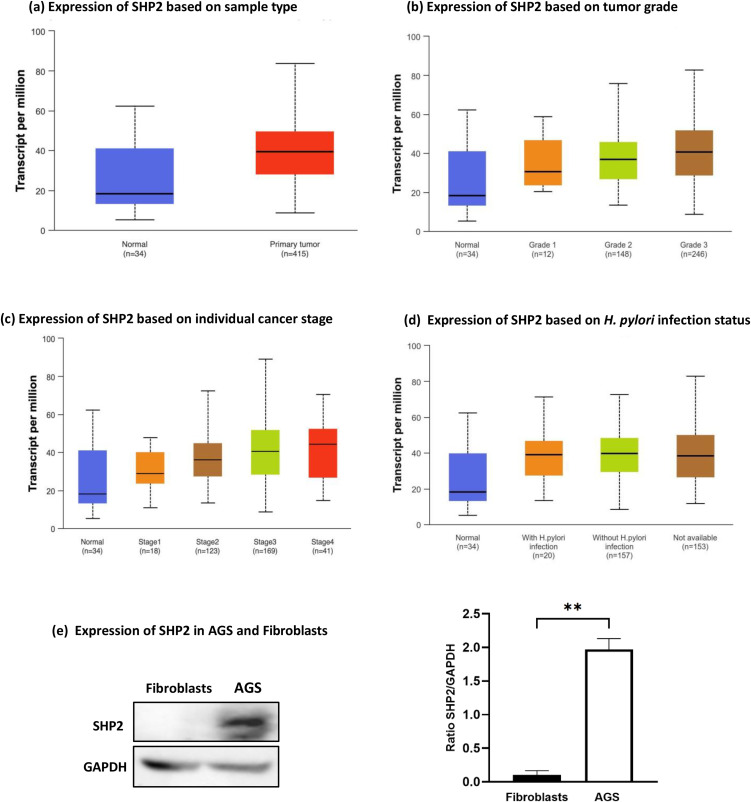
The expression of SHP2 in stomach adenocarcinoma (STAD) specimens. Panel (a) illustrates the expression levels of SHP2 in normal tissues (n = 34) compared to primary STAD samples (n = 415). Panel (b) presents SHP2 expression across different cancer grades (stages 1–4) of STAD. Panel (c) examines the expression levels of SHP2 across various cancer stages in STAD, juxtaposed with the normal group. Panel (d) shows the expression levels of SHP2 in STAD with and without *H. pylori* infection, compared to the normal group. Panel (e) features a Western blot that depicts both SHP2 and GAPDH (as a loading control) in AGS gastric cancer cell lines compared to control fibroblasts; the quantification of protein expression is shown using Image Lab software, with results presented as mean ± SD from two independent biological replicates. Significant findings are indicated by asterisks (**P < 0.01). Original blots can be viewed in Supplemental Raw Blot Images B1.

In our analysis utilizing UALCAN, we examined the gene expression data from the stomach adenocarcinoma (STAD) section of The Cancer Genome Atlas (TCGA) using 449 sample sections distributed over 34 normal sections and 415 primary tumor section; the tool is accessible at http://ualcan.path.uab.edu/cgi-bin/. Although the primary focus of this study is SHP2, it was also essential to assess the expression of RTKs due to their strong functional relationship with SHP2. SHP2 mediates intracellular signaling cascades triggered by RTK activation, primarily through the Ras/MAPK and PI3K/AKT pathways, playing a key role in cell proliferation, differentiation and survival [19,20]. As shown in Table 1, 22 out of 49 RTKs were upregulated (P < 0.05) and 6 were downregulated in gastric cancer compared to normal cells (P < 0.05), with 21 showing no significant change. Furthermore, SHP2 (coded by PTPN11 gene) was found to be upregulated in STAD (Fig 2a) regardless of the cancer grades (Fig 2b) and stages (Fig 2c) (P < 0.05). Further analysis through Western blotting confirmed elevated levels of SHP2 in AGS gastric cancer cell lines compared to fibroblasts (P < 0.05), as demonstrated in Fig 2e.

Our data also explored the influence of *H. pylori* infection on gene expression within STAD. Interestingly, as shown in Table 1 and Fig 2d, the expression of PTPN11 (coding for SHP2) did not significantly differ between tumors with or without *H. pylori* infection, suggesting minimal impact of the infection on this gene’s expression in STAD. This observation is consistent with the well-established mechanistic basis of CagA-mediated SHP2 activation, which operates at the post-translational rather than transcriptional level. Upon translocation into gastric epithelial cells, CagA directly binds to the SH2 domains of SHP2 and induces conformational activation of its phosphatase activity without necessitating upregulation of PTPN11 gene expression [10,11]. Furthermore, the strength of this interaction has been shown to depend on the structural polymorphism of CagA, particularly the number and sequence context of its EPIYA motifs, which govern binding affinity and downstream SHP2 activation in a strain-dependent manner [12,13]. Taken together, these findings indicate that the oncogenic contribution of SHP2 in *H. pylori*-associated gastric carcinogenesis is a post-translational, structure-dependent phenomenon that is not captured by transcriptomic profiling and therefore does not diminish the biological and therapeutic relevance of targeting SHP2 in gastric cancer irrespective of *H. pylori* infection status. Conversely, genes such as EGFR and ERBB3 were notably downregulated in the presence of *H. pylori*, hinting at potential specific disruptions caused by the infection. Moreover, a comparison of gene expressions between normal and tumor tissues revealed a general trend of upregulation in numerous genes within tumors, indicative of the aggressive nature and altered metabolic states of cancerous cells. In contrast, genes like EPHA6, EPHA7, NTRK3 and ALK were consistently downregulated, which may suggest their roles as tumor suppressors or their involvement in particular dysregulated pathways.

The RTKs analyzed include: EGFR (Epidermal Growth Factor Receptor); ERBB2–4 (Erb-B2 Receptor Tyrosine Kinase 2–4); INSR (Insulin Receptor); IGF1R (Insulin-Like Growth Factor 1 Receptor); INSRR (Insulin Receptor-Related Receptor); FGFR1–4 (Fibroblast Growth Factor Receptor 1–4); PDGFRA-B (Platelet-Derived Growth Factor Receptor Alpha- Beta); FLT1 (Fms-Related Tyrosine Kinase 1); KDR (Kinase Insert Domain Receptor, also known as VEGFR-2); FLT4 (Fms-Related Tyrosine Kinase 4); FGFRL1 (Fibroblast Growth Factor Receptor-Like 1); EPHA1–8 (EPH Receptor A1-8); EPHA10 (EPH Receptor A10); EPHB1–4 (EPH Receptor B1-4); EPHB6 (EPH Receptor B6); AXL (AXL Receptor Tyrosine Kinase); MERTK (MER Proto-Oncogene, Tyrosine Kinase); TYRO3 (TYRO3 Protein Tyrosine Kinase); TIE1 (Tyrosine Kinase with Immunoglobulin-Like and EGF-Like Domains 1); TEK (TEK Receptor Tyrosine Kinase, also known as TIE2); RET (Ret Proto-Oncogene); NTRK1–3 (Neurotrophic Receptor Tyrosine Kinase 1–3); RYK (Receptor-Like Tyrosine Kinase); DDR1 (Discoidin Domain Receptor Tyrosine Kinase 1); DDR2 (Discoidin Domain Receptor Tyrosine Kinase 2); MET (MET Proto-Oncogene, Receptor Tyrosine Kinase); MST1R (Macrophage Stimulating 1 Receptor, also known as RON); LTK (Leukocyte Receptor Tyrosine Kinase); ALK (Anaplastic Lymphoma Kinase); ROS1 (ROS Proto-Oncogene 1, Receptor Tyrosine Kinase). PTPN11 (Tyrosine-protein phosphatase non-receptor type 11). Gene expression designations are based on UALCAN platform Wilcoxon rank-sum test statistical outputs. (*) indicates statistically significant change at P < 0.05 as reported by the platform. Insignificant change denotes P ≥ 0.05. As UALCAN does not provide raw expression values or exact p-values, directional designations with significance notation represent the maximum level of quantitative transparency achievable from this platform.

### Inhibitory effects of NSC 57774 on AGS gastric cancer cell proliferation

Given the observed upregulation of SHP2 in gastric cancer and its role in receptor tyrosine kinase (RTK) signaling, we recognized the potential promise of investigating the impact of inhibiting this enzyme. NSC 57774, a SHP2 inhibitor previously identified by our research group as an anticancer agent in breast cancer [14], was thus selected for evaluation in gastric cancer. Consequently, we embarked on assessing the effect of inhibiting SHP2 on tumor proliferation in gastric cancer. In this study, the anti-proliferative effects of NSC 57774 and the well-established anticancer agent doxorubicin on AGS gastric cancer cell lines were evaluated. IC50 values were determined using the MTT assay at three time points: 24, 48 and 72 hours. The results, as summarized in Table 2, reveal that NSC 57774 exhibited IC50 values of 2.7μM, 1.37μM and 0.86μM at 24, 48 and 72 hours, respectively. Similarly, DOX demonstrated IC50 values of 0.12μM, 0.034μM and 0.039μM at the corresponding time intervals. For the SHP2 inhibitor positive control, NSC 87877 was used at a dosage of 70µM, equivalent to its IC50 value determined prior to this experiment by MTT assay (S1 Fig) (Table 2). Notably, the IC50 value of NSC 57774 on fibroblasts was higher than that observed on AGS cells, indicating a lower cytotoxicity of NSC 57774 towards fibroblasts. This suggests that NSC 57774 exhibits selective toxicity against AGS cells, while demonstrating minimal toxicity to non-cancerous cells (S2 Fig). Based on the IC50 findings, we selected to assess the AGS proliferation against 3 uM of NSC 57774, to explore the effects at a double IC50 dose and to facilitate a comparative analysis. For Doxorubicin, we opted to use a concentration of 0.034 μM, corresponding to the IC50 value at 48 hours, for our subsequent molecular studies. For the SHP2 positive control, the selected tested concentration was based on the obtained IC50 values (Table 2) which was equivalent to 70 μM.

**Table 2:** IC₅₀ values of NSC 57774, NSC 87877 and Doxorubicin against gastric cancer cells (AGS) following 24, 48 and 72 hours of treatment.

IC50 (μM)			
**Treatment Duration**	**AGS +** **NSC 57774**	**AGS +** **NSC 87877**	**AGS +** **Doxorubicin**
**24h**	2.7	>70*	0.12
**48h**	1.37	70.58	0.034
**72h**	0.86	74.07	0.039

* IC50 could not be detected at 24 h.

**Fig 3:**
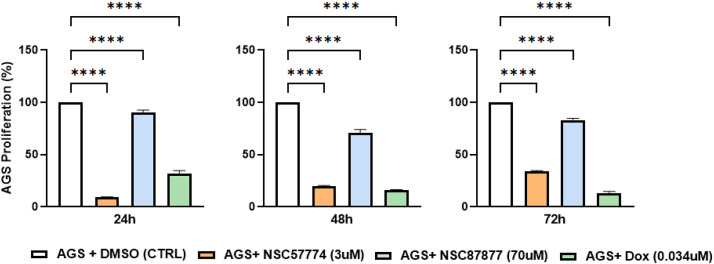
Effect of NSC 57774 on AGS gastric cancer cell line proliferation. The percentage proliferation for AGS cell lines, untreated or treated with 3 µM NSC 57774, was determined by MTT assay at 24, 48 and 72 hours. Additionally, AGS cells were treated with the SHP2 inhibitor NSC 87877 at 70 µM (SHP2 positive control) and the anticancer agent Dox at 0.034 µM as controls. Significant results were denoted by asterisks ****P < 0.0001). The percentage of AGS proliferation was presented as the mean ± standard deviation (SD) of two independent experiments.

All statistical comparisons were performed as described in the Statistical Analysis section of the Materials and Methods; briefly, Student’s unpaired t-test was applied for comparisons against the DMSO vehicle control, and One-Way ANOVA with Dunnett’s multiple comparisons test was used for inter-group comparisons. As shown in Fig 3, treatment of AGS cell lines with 3 µM NSC 57774 for 24 hours resulted in a significant reduction in cell proliferation to 9.12%, compared to the control group (P < 0.001). This suppressive effect of NSC 57774 was maintained at 48 and 72 hours, with proliferation decreasing to 19.55% and 33.32%, respectively (P < 0.001). In parallel, doxorubicin, used as a control anticancer agent at 0.034 µM, also significantly reduced AGS cell proliferation. Intriguingly, the used control SHP2 inhibitor NSC 87877 (SHP2 positive control), at a dosage of 70µM, equivalent to the IC50 value determined prior to this experiment by MTT assay (S1 Fig) (Table 2), diminished AGS cell proliferation as well, but very less effectively when compared to NSC 57774.

### Migration ability of AGS cells decreases after inhibiting SHP2 with NSC 57774

**Fig 4:**
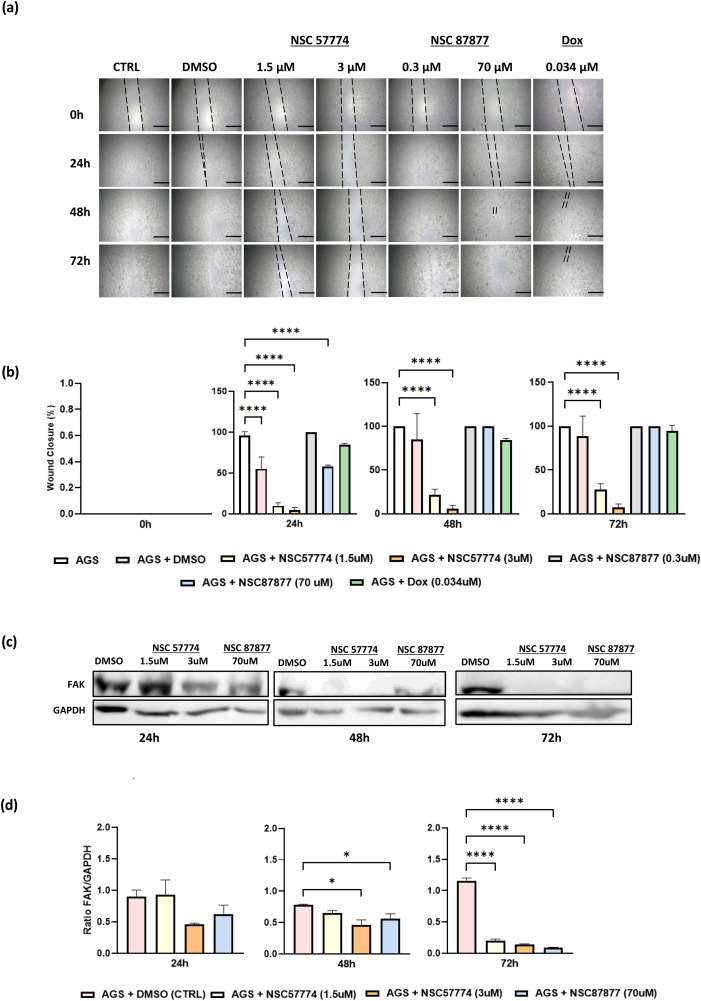
Effect of NSC 57774, NSC 87877 and Doxorubicin on gastric cancer cell migration. (a) Representative images from the wound healing assay of AGS cell lines treated with 1.5µM NSC 57774, 3µM NSC 57774, 0.3µM NSC 87877, 70µM NSC 87877, 0.034µM Dox and the vehicle control (DMSO) for 24, 48 and 72 hours. (b) The percentage of wound closure achieved by migrated cells was quantified using the wound healing size tool in ImageJ and is presented as the mean ± standard deviation (SD) of two independent experiments. The scale bar represents 400μm. (c) Western blots showing FAK expression with GAPDH used as a loading control. AGS cells were treated with NSC 57774 at concentrations of 1.5 µM and 3 µM and NSC 87877 at 70 µM, compared to a control treated with DMSO, across time intervals of 24, 48 and 72 hours. (d) Protein expression levels were quantified using Image Lab software and are presented as the mean ± SD from two independent experiments. Statistical significance is indicated by asterisks: *P < 0.05, **P < 0.01, ***P < 0.001. The blot represents one representative biological replicate selected for clarity of presentation, while quantification bar graphs reflect mean ± SD from two independent biological replicates. Blots from 4c were cropped from the same membrane. Full uncropped blots are provided in Supplementary Raw Blot Images B2.

In this study, we investigated the effects of various treatments on the migration capabilities of AGS gastric cancer cell lines, employing a wound healing assay. Based on the IC50 findings, we selected two concentrations of NSC 57774 for further study: 1.5 μM and/or 3 μM. These concentrations were chosen to approximate the IC50 value observed at 48 hours and to explore the effects at a double dose, facilitating a comparative analysis. For Dox, we opted to use a concentration of 0.034 μM, corresponding to the IC50 value at 48 hours, for our subsequent molecular studies. For the SHP2 inhibitor NSC 87877, two concentrations were tested: 0.30 µM and 70 µM. Its worthy to mention that the 0.3µM concentration of NSC 87877 was selected based on the manufacturer (MedChemExpress) and the literature where the IC50 was detected at this concentration for most cancer cells other than AGS cells [21]. Therefore, the cell lines were treated with two concentrations of NSC 57774 (1.5 µM and 3 µM), NSC 87877 (0.3 µM and 70 µM), Doxorubicin (0.034 µM), or the vehicle control (DMSO) and assessed at intervals of 24, 48 and 72 hours. As depicted in Fig 4A, treatments with 1.5 µM and 3 µM of NSC 57774 resulted in a time-dependent decrease in the migration capability of these cell lines. Comparative analysis further demonstrated a significant reduction in the migrated area for cells treated with NSC 57774, with the inhibitory effect being more pronounced at the higher concentration of 3 µM compared to 1.5 µM (Fig 4B). The analysis confirmed a statistically significant reduction in cell migration at both concentrations of NSC 57774 and with 0.034 µM of Doxorubicin compared to the vehicle-treated controls (DMSO) after 24h, 48 and 72h of treatment. For NSC 57774 (70µM), statistically significant reduction in cell migration was observed only after 24h and 48h of treatment. Notably, the inhibitory effect of NSC 57774 on cellular migration was more pronounced than that observed with both NSC 87877 and Doxorubicin, highlighting the potential of NSC 57774 as a superior inhibitor in the context of AGS cell line migration (Fig 4B). To further investigate the effects of NSC 57774 on cell migration, we performed Western blot analyses to assess FAK expression levels following treatment with NSC 57774 (1.5 µM and 3 µM), NSC 87877 (70 µM) and DMSO as a control. These experiments were conducted at three time points: 24, 48 and 72 hours. As shown in Figs 4C and 4D, our results demonstrate a consistent decrease in FAK levels with NSC 57774 at 3 µM and with NSC 87877 at all-time points, as well as with NSC 57774 at 1.5 µM at 48 and 72 hours.

### Effect of SHP2 inhibition by NSC 57774 on ERK, AKT and STAT3 signaling pathways

**Fig 5:**
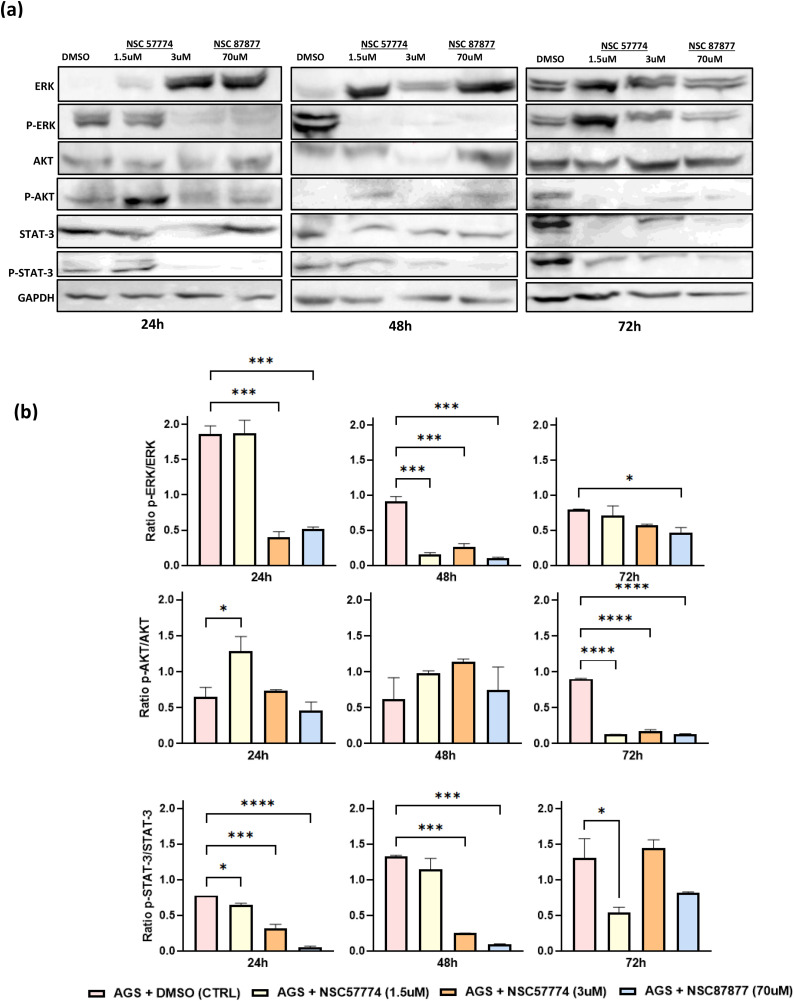
Effects of NSC 57774 on key signaling pathways in AGS cells over 24, 48 and 72 hours. SHP2 is a central mediator of oncogenic signaling downstream of receptor tyrosine kinases (RTKs), promoting tumor cell survival, proliferation, and metastasis through activation of the MAPK/ERK, PI3K/AKT, and JAK/STAT3 cascades (a) Western blots showing phosphorylated and non-phosphorylated forms of SHP2 downstream effectors (ERK, AKT and STAT3), with GAPDH used as a loading control. AGS cells were treated with NSC 57774 at concentrations of 1.5 µM and 3 µM, NSC 87877 at 70 µM, compared to a control treated with DMSO, across time intervals of 24, 48 and 72 hours. (b) Quantification of the phosphorylated-to-total protein ratios (p-ERK/ERK, p-AKT/AKT, and p-STAT3/STAT3) reflects the degree of pathway activation or suppression following treatment. Protein expression levels were quantified using ImageJ and are presented as the mean ± SD from two independent experiments. Statistical significance is indicated by asterisks: *P < 0.05, **P < 0.01, ***P < 0.001. The blot represents two biological replicates, while quantification bar graphs reflect mean ± SD from two independent biological replicates. Blots from 5a were cropped from the same membrane. Full uncropped blots are provided in Supplementary Raw Blot Images B3.

To elucidate the mechanisms underlying the observed attenuation of cancer cell proliferation and migration following SHP2 inhibition, we analyzed the effects of SHP2 inhibitors NSC 57774 and NSC 87877 on key signaling molecules ERK, AKT and STAT3 in gastric cancer cells across treatment intervals of 24, 48 and 72 hours. These molecules play pivotal roles in gastric cancer, influencing cellular growth, survival and metastatic potential [22]. As shown in Fig 5, treatment with NSC 57774 at concentrations of 3 µM and NSC 87877 at 70 µM, led to a time-dependent decrease in the p-ERK/ERK ratio in gastric cancer cells. At 24 hours, NSC 57774 at 1.5 µM did not produce a statistically significant reduction in p-ERK levels compared to the DMSO control, whereas NSC 57774 at 3 µM and NSC 87877 at 70 µM resulted in a significant decrease (p < 0.001). Notably, the 3 µM dose of NSC 57774 demonstrated an effect comparable to that of NSC 87877 at 70 µM, underscoring its significant potency. At 72 hours, only NSC 87877 at 70 µM maintained a statistically significant reduction in p-ERK levels (p < 0.05), while the effect of NSC 57774 at both doses did not reach statistical significance at this timepoint. In terms of AKT phosphorylation (Fig 5), an initial significant (p < 0.05) increase in the p-AKT/AKT ratio was observed 24 hours after initiating treatment with NSC 57774 at 1.5 µM, suggesting an early and transient activation of AKT signaling which may be a direct response to SHP2 inhibition. Interestingly, this effect was not maintained as by 48 hours, no significant changes in p-AKT/AKT levels were evident across all treatments. By 72 hours, a highly significant (p < 0.0001) significant reduction in p-AKT/AKT levels was noted across all treatment groups. Furthermore, treatment with NSC 57774 at 3 µM and NSC 87877 at 70 µM resulted in a consistent decrease in p-STAT3/STAT-3 (Fig 5) levels at 24 and 48 hours. However, a notable divergence was observed at 72 hours, where NSC 57774 at 1.5 µM showed an unexpected increase in p-STAT3 levels above the control, while NSC 57774 at 3 µM did not reach statistical significance. Only NSC 87877 at 70 µM maintained a statistically significant reduction in p-STAT3/STAT-3 levels at this timepoint (p < 0.05), suggesting that sustained inhibition of STAT3 phosphorylation may require higher inhibitory potency over prolonged treatment periods.

### Effect of SHP2 inhibition by NSC 57774 on apoptosis and inflammatory response

**Fig 6:**
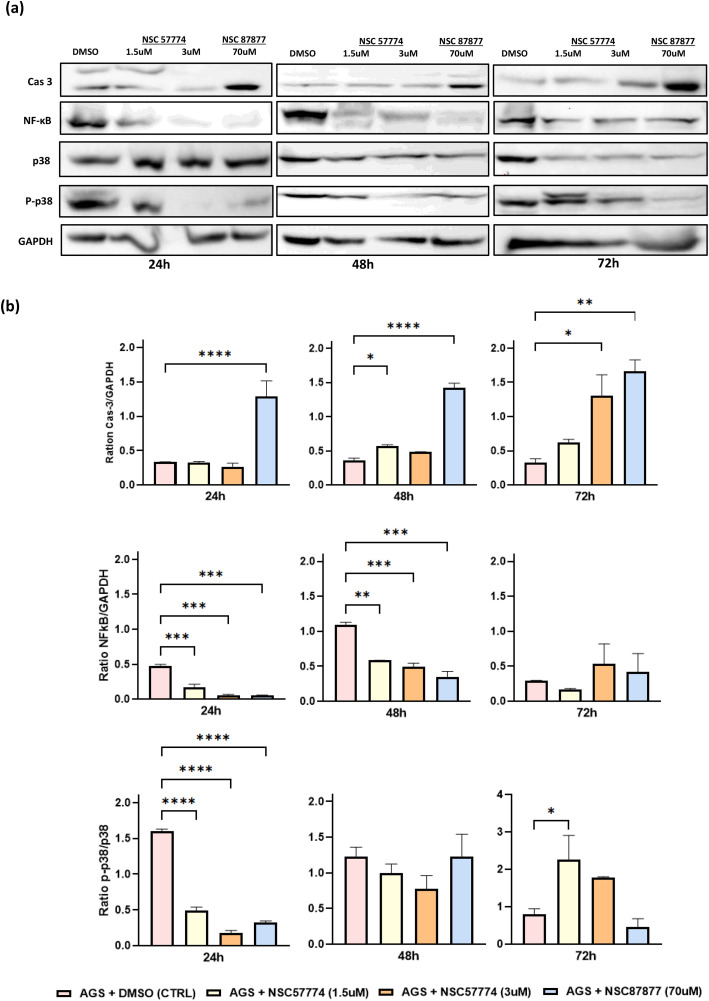
Effects of NSC 57774 on Apoptosis and inflammatory response in AGS cells over 24, 48 and 72 hours. SHP2 inhibition has been linked to the modulation of key apoptotic and inflammatory mediators; cleaved caspase-3 serves as a terminal effector of apoptosis, NF-κB is a master regulator of pro-inflammatory and pro-survival signaling, and p38 MAPK represents a stress-activated kinase involved in both inflammatory response and apoptotic regulation downstream of cellular stress signals. (a) Western blots showing cleaved caspase 3, NF-κB, phosphorylated and non-phosphorylated forms of p38 with GAPDH used as a loading control. AGS cells were treated with NSC 57774 at concentrations of 1.5 µM and 3 µM and NSC 87877 at 70 µM, compared to a control treated with DMSO, across time intervals of 24, 48 and 72 hours. (b) Quantification of cleaved caspase-3 expression, NF-κB levels, and the p-p38/p38 ratio reflects the degree of apoptotic induction, inflammatory suppression, and stress pathway activation following treatment, respectively. Protein expression levels were quantified using ImageJ and are presented as the mean ± SD from two independent experiments. Statistical significance is indicated by asterisks: *P < 0.05, **P < 0.01, ***P < 0.001. The blot represents two biological replicates, while quantification bar graphs reflect mean ± SD from two independent biological replicates. Blots from 6a were cropped from the same membrane. Full uncropped blots are provided in Supplementary Raw Blot Images B4.

To investigate the effects of NSC 57774 on apoptosis and inflammatory response, we conducted western blot analyses for cleaved caspase-3, NF-κB and phospho-p38 using NSC 57774 (1.5 µM and 3 µM), as well as NSC 87877 (70 µM) and DMSO as control. These experiments were performed at three different time points: 24, 48 and 72 hours. As shown in Fig 6, our results revealed that at 24 hours, only NSC 87877 at 70 µM induced a significant increase in cleaved caspase-3 levels (p < 0.0001), while NSC 57774 at both 1.5 µM and 3 µM did not produce statistically significant changes at this timepoint. At 48 hours, NSC 87877 continued to drive a significant increase in cleaved caspase-3 levels (p < 0.0001), with NSC 57774 at 1.5 µM showing only a modest and marginally significant elevation (p < 0.05). By 72 hours, cleaved caspase-3 levels were significantly elevated across all treatment groups, with NSC 57774 at 1.5 µM (p < 0.05), NSC 57774 at 3 µM (p < 0.05), and NSC 87877 at 70 µM (p < 0.01) all demonstrating significant increases compared to the DMSO control. Interestingly, NF-κB expression levels consistently decreased at 24 and 48 hours across all concentrations and time points for all treatments suggesting a promising role of NSC 57774 as reducing inflammation. However, at 72 hours, the reduction in NF-κB levels did not reach statistical significance for any of the treatment groups, suggesting that the anti-inflammatory effect of these compounds may attenuate over prolonged treatment periods. Phospho-p38 expression levels significantly decreased after 24 hours with NSC 57774 at 1.5 µM and 3 µM (p < 0.0001), while NSC 87877 at 70 µM did not reach statistical significance at this timepoint. The expression level showed no significant differences after 48 hours across all treatment Surprisingly, at 72 hours, only NSC 57774 at 1.5 µM showed a significant increase in phospho-p38 expression levels (p < 0.05), while NSC 57774 at 3 µM and NSC 87877 at 70 µM did not reach statistical significance at this timepoint.

## Discussion

Our findings reveal marked alterations in the expression of receptor tyrosine kinases (RTKs) and the non-receptor protein tyrosine phosphatase SHP2 in stomach adenocarcinoma using the UALCAN platform and The Cancer Genome Atlas (TCGA) data. Notably, 24 out of 48 RTKs were found to be upregulated in cancerous versus normal gastric cells [15,23]. Concomitantly, SHP2 was also upregulated, as evidenced by TCGA data, and confirmed by Western blot analysis. The simultaneous upregulation of RTKs and SHP2 suggests a synergistic oncogenic interaction, further emphasizing the pivotal role of SHP2 in the molecular pathogenesis of gastric cancer. These findings underscore the therapeutic potential of targeting the RTK-SHP2 signaling axis in the treatment of stomach adenocarcinoma. The TCGA consortium classifies gastric adenocarcinoma into four molecular subtypes: EBV-positive, MSI-high, genomically stable (GS), and chromosomal instability (CIN) [24]. Among these, the CIN subtype representing approximately 50% of STAD cases and predominantly comprising intestinal-type tumors which exhibits the most pronounced RTK amplification and constitutive RAS/MAPK/PI3K-AKT pathway activation, making it the subtype most likely to benefit from SHP2-targeted therapy. The broad upregulation of RTKs and SHP2 observed in our TCGA/UALCAN analysis is therefore most consistent with CIN-subtype predominance in the dataset. Importantly, the AGS cell line used in this study is a well-established model of intestinal-type gastric adenocarcinoma, directly representative of the CIN subtype, providing additional rationale for its selection. In contrast, EBV-positive tumors are predominantly driven by PI3K rather than RAS/MAPK signaling, while GS tumors are driven by CDH1/RHOA mutations with comparatively low RTK dependence, subtypes in which SHP2 monotherapy may show limited efficacy. MSI-high tumors, characterized by high mutational burden and PD-L1 overexpression, represent the subpopulation most likely to benefit from combining SHP2 inhibitors with immune checkpoint blockade, as recently demonstrated by Kang et al. [25]. Future subtype-stratified analyses and multi-cell-line validation studies will be essential to fully delineate the therapeutic spectrum of NSC 57774 across the molecular landscape of gastric adenocarcinoma [22,24,25]. The significance of SHP2 upregulation in gastric cancer is twofold. First, SHP2 plays a critical role in transducing signals from activated RTKs to downstream pathways, including the Ras/MAPK pathway, essential for cell proliferation, survival and differentiation [4]. The overexpression of RTKs, coupled with the enhanced activity of SHP2, likely contributes to the hyperactivation of these pathways, promoting oncogenic transformations and tumor progression [5]. Second, SHP2 influences several key cellular processes beyond RTK signaling, impacting cell migration and apoptosis, which are crucial for cancer metastasis and survival [6]. When examining the impact of *H. pylori* infection on stomach adenocarcinoma, the data reveals that most genes show insignificant changes in expression, suggesting that the presence of the bacteria does not significantly alter gene expression within established tumors [9]. Importantly, the absence of significant transcriptional changes in PTPN11 between *H. pylori*-positive and *H. pylori*-negative STAD specimens does not negate the functional role of SHP2 in *H. pylori*-associated gastric carcinogenesis. The oncogenic activity of CagA might be mediated through direct protein-protein interaction with SHP2, leading to constitutive phosphatase activation, which is a post-translational phenomenon not captured by transcriptomic profiling. However, specific genes like EGFR and ERBB3 exhibit downregulation in the presence of *H. pylori*, potentially pointing to specific pathways disrupted by the infection [9]. Additionally, comparisons between normal and tumor tissues both with and without *H. pylori* infection underscore that many RTK genes are upregulated in tumors, emphasizing the aggressive nature of cancer cells and their enhanced metabolic or proliferative states [26]. Intriguingly, genes such as EPHA6, EPHA7 and ALK are consistently downregulated across these comparisons, possibly suggesting roles as tumor suppressors or specific dysregulation in these pathways [8]. Diving deeper into specific patterns, the EGFR and ERBB family genes demonstrate notable changes; EGFR, for example, is specifically downregulated when *H. pylori* is present, which is significant considering its role in cell proliferation and survival [3]. This could indicate a unique interaction between *H. pylori* and the pathways mediated by EGFR. The EPHA family also shows variability, with genes like EPHA8 being upregulated in the presence of *H. pylori* and downregulated in its absence, highlighting the complex roles these genes may play in tumor biology and their interaction with the infection [5].

These observations lead to several conclusions. While *H. pylori* infection does alter the expression of some genes within tumors, the majority of gene expression changes are primarily driven by the presence of the tumor itself rather than the infection [27]. This insight opens up potential targeted therapy opportunities, particularly as understanding which genes are consistently upregulated (such as MET and PDGFRB) or specifically altered by *H. pylori* infection (like EGFR and ERBB3) could lead to the development of specific therapeutic strategies for treating stomach adenocarcinoma, with or without the presence of *H. pylori* [9]. Given the observed upregulation of SHP2 in gastric cancer, we recognized the potential of targeting this enzyme for therapeutic intervention. NSC 57774, a SHP2 inhibitor previously identified by our research group as an anticancer agent in breast cancer [14], was selected for evaluation in gastric cancer. Our study indicates a notable anti-proliferative effect of NSC 57774 on AGS gastric cancer cell lines, highlighting the potential therapeutic value of SHP2 inhibition in gastric cancer treatment. The time-dependent decrease in cell proliferation observed with NSC 57774 treatment aligns with the upregulation of SHP2 in gastric cancer pathogenesis, suggesting that SHP2 plays a significant role in tumorigenesis and may be a viable target for pharmacological intervention [6]. The therapeutic relevance of SHP2 inhibition in gastric cancer continues to gain momentum in the recent literature. Zheng et al. demonstrated that SHP2 inhibition effectively mitigates adaptive resistance to MEK inhibitors in KRAS-mutant gastric cancer through suppression of KSR1 activity, underscoring the rationale for SHP2-targeted combination strategies [28]. Interestingly, while Doxorubicin remains a cornerstone in cancer therapy, our findings suggest that NSC 57774 may exhibit superior potency in reducing gastric cancer cell viability, evidenced by its lower IC50 values. This positions NSC 57774 as a potentially more effective agent against SHP2-expressing tumors [26]. Moreover, the comparative analysis with NSC 87877 further supports the specificity of NSC 57774’s action. Although NSC 87877 did reduce AGS cell proliferation, its effect was less pronounced than that of NSC 57774, highlighting the latter’s enhanced inhibitory activity. This differential efficacy may be attributed to the structural and molecular distinctions between the two compounds, which could influence their binding affinities and subsequent intracellular effects [8]. It is important to note that Doxorubicin was included solely as a standard cytotoxic reference agent rather than a mechanistic comparator, given its fundamentally distinct mode of action via DNA intercalation and topoisomerase II inhibition. The superior IC50 values of NSC 57774 relative to Doxorubicin should therefore be interpreted in the context of its targeted SHP2-specific activity, further underscoring its potential as a precision therapeutic agent for SHP2-expressing gastric tumors. Regarding cell migration, the results show a time-dependent decrease in migration capability with increasing concentrations of NSC 57774 (1.5 µM and 3 µM), indicating a strong dose-response relationship [6]. This suggests that NSC 57774 effectively inhibits cellular mechanisms involved in migration, which are crucial for cancer progression and metastasis. The more pronounced inhibitory effect at 3 µM compared to 1.5 µM underscores the potential of higher doses of NSC 57774 in more aggressively curbing cancer cell movement. Interestingly, NSC 57774 displayed superior inhibitory effects on cell migration compared to NSC 87877 and Doxorubicin. This suggests that NSC 57774 might interact with cellular pathways more effectively than NSC 87877, despite both being SHP2 inhibitors [27]. The difference in efficacy between these two inhibitors could be attributed to variations in molecular structure, affinity for the SHP2 enzyme, or their ability to interfere with additional molecular targets involved in migration processes. Doxorubicin, known primarily for its cytotoxic effects rather than as an inhibitor of migration, showed the least effect on migration among the treatments. This aligns with its mode of action, which primarily involves DNA intercalation and the generation of free radicals, leading to cell death rather than specifically inhibiting cell movement [26]. This comparison highlights the potential of targeted therapies like NSC 57774, which may offer advantages over traditional chemotherapeutic agents like Doxorubicin in specific cancer treatment contexts, particularly where inhibition of migration and metastasis is desired. An inherent limitation of the wound healing assay employed in this study is its inability to fully dissociate anti-migratory effects from anti-proliferative effects, as both processes contribute to wound closure. The inclusion of mitomycin C pre-treatment to selectively inhibit cell division and isolate directional cell migration was not feasible in the current study due to reagent unavailability at the time of revision. Future studies will incorporate this control to provide a more mechanistically precise characterization of the anti-migratory activity of NSC 57774.To elucidate the mechanisms underlying the observed attenuation of cancer cell proliferation and migration following SHP2 inhibition, we analysed the effects of SHP2 inhibitors NSC 57774 and NSC 87877 on key signaling molecules (ERK, AKT and STAT3) in gastric cancer cells across treatment intervals of 24, 48 and 72 hours. These molecules play pivotal roles in gastric cancer, influencing cellular growth, survival and metastatic potential [6]. The p-ERK/ERK ratio decreased in a time-dependent manner upon treatment with NSC 57774 at 3 µM and NSC 87877 at 70 µM. This reduction suggests effective inhibition of the ERK signaling pathway, which is crucial for cell proliferation and survival [6]. At 24 hours, NSC 57774 at 1.5 µM did not produce a statistically significant reduction in p-ERK levels, suggesting that a higher concentration threshold is required for meaningful ERK pathway suppression at early timepoints. The comparable effects of the 3 µM dose of NSC 57774 and NSC 87877 underscore the potential of NSC 57774 as a potent SHP2 inhibitor at higher concentrations. At 72 hours, only NSC 87877 at 70 µM maintained statistically significant ERK suppression, while both doses of NSC 57774 failed to reach significance at this timepoint. The rebound in p-ERK levels at 72 hours with NSC 57774 at both doses may indicate transient efficacy or adaptive resistance mechanisms in the cancer cells, highlighting the need for continuous or combination therapy strategies to maintain suppression of ERK activation [26].Concerning AKT, an initial significant (p < 0.05) increase in the p-AKT/AKT ratio was observed at 24 hours post-treatment with NSC 57774 at 1.5 µM. No significant changes were noted at 48 hours across all treatments. However, by 72 hours, a highly significant (p < 0.0001) reduction in p-AKT/AKT levels was noted across all treatment groups. The initial rise in p-AKT levels suggests a compensatory feedback activation of AKT signaling following SHP2 inhibition, potentially as a survival mechanism by the cancer cells. The subsequent highly significant decrease in p-AKT levels by 72 hours across all treatments might reflect the exhaustion of compensatory pathways or effective downstream inhibition by the SHP2 inhibitors. This biphasic response highlights the complexity of cellular signaling responses to SHP2 inhibition and suggests potential timing strategies for therapeutic interventions to maximize the efficacy of treatment [6]. A consistent decrease in p-STAT3/STAT3 levels was observed with NSC 57774 at 3 µM and NSC 87877 at 70 µM at 24 and 48 hours. However, a notable divergence emerged at 72 hours, where NSC 57774 at 1.5 µM showed an unexpected elevation in p-STAT3 levels above the control, and NSC 57774 at 3 µM did not reach statistical significance. Only NSC 87877 at 70 µM maintained a statistically significant reduction in p-STAT3/STAT3 at this timepoint (p < 0.05). The reduction in p-STAT3 levels indicates successful inhibition of STAT3 signaling, which is crucial for various oncogenic processes including tumorigenesis, immune evasion and inflammation. The dose-dependent response of NSC 57774 suggests its efficacy is concentration-sensitive, with higher doses achieving more pronounced and sustained inhibition. The paradoxical increase in p-STAT3 levels observed at 72 hours with NSC 57774 at 1.5 µM may reflect the activation of compensatory feedback mechanisms following prolonged SHP2 inhibition at sub-optimal concentrations, further underscoring the necessity for either higher dosing or prolonged treatment durations to achieve consistent therapeutic effects on STAT3 phosphorylation [26]. The results of our study provide significant insights into the molecular mechanisms underlying the effects of the SHP2 inhibitors NSC 57774 and NSC 87877 on apoptosis, cell survival, migration and inflammatory response. At 24 and 48 hours, only NSC 87877 at 70 µM induced a significant increase in cleaved caspase-3 levels, while NSC 57774 at both doses did not produce statistically significant changes until 72 hours, at which point all treatment groups demonstrated significant elevations in cleaved caspase-3. This suggests effective inhibition of SHP2, which is known to promote cell survival and proliferation. This pattern indicates a concentration- and time-dependent induction of apoptosis, where NSC 87877 engages apoptotic pathways more rapidly, while NSC 57774 requires prolonged exposure to achieve comparable pro-apoptotic effects [8].

NF-κB, a transcription factor involved in inflammatory responses and cell survival, consistently decreased across all treatments at 24 and 48 hours This suggests that both NSC 57774 and NSC 87877 effectively suppress inflammatory signaling pathways, which is consistent with SHP2’s role in regulating NF-κB activity [8]. However, at 72 hours, the reduction in NF-κB levels did not reach statistical significance for any treatment group, suggesting that the anti-inflammatory effect of these compounds may attenuate over prolonged treatment periods, possibly due to compensatory re-activation of inflammatory signaling pathways. The significant decrease in p-p38 levels after 24 hours with NSC 57774 at 1.5 µM and 3 µM (p < 0.0001) indicates an immediate response to SHP2 inhibition affecting the p38 MAPK pathway. Notably, NSC 87877 at 70 µM did not reach statistical significance at this timepoint, suggesting differential engagement of the p38 pathway between the two inhibitors at early treatment stage. After 72 hours, only NSC 57774 at 1.5 µM showed a significant increase in p-p38 levels (p < 0.05), while NSC 57774 at 3 µM and NSC 87877 did not reach significance, suggesting that NSC 57774 might be engaging in feedback loops or alternative pathways that upregulate p-p38 over prolonged exposure at lower concentrations [8].

The paradoxical increase in phospho-p38 levels observed at 72 hours specifically with NSC 57774 at 1.5 µM warrants careful interpretation. We propose two non-mutually exclusive explanations: First, p38 MAPK activation at later time points may represent a compensatory stress response to prolonged SHP2 inhibition and associated cellular damage. SHP2 is known to regulate multiple phosphatase substrates, and its sustained inhibition can trigger adaptive stress signaling through alternative MAPK branches [29]. Second, the late-phase p38 activation may contribute to additional pro-apoptotic signaling, as p38 has well-established roles in both inflammatory and apoptotic pathways, depending on cellular context and activation duration [30]. Notably, the concomitant increase in cleaved caspase-3 at 72 hours is consistent with this interpretation. Whether this late p38 activation represents a therapeutic limitation or an auxiliary apoptotic mechanism requires further investigation, which we have now identified as a specific future research direction.

A key limitation of the present study is its restriction to in vitro experimentation using a single gastric cancer cell line. While the AGS cell line is well-established and widely validated for mechanistic proof-of-concept investigations, it does not fully recapitulate the complexity of the in vivo tumour microenvironment, including immune cell interactions, stromal components, and tumour heterogeneity. Nevertheless, the selection of AGS cells was not arbitrary but was underpinned by a strong biological rationale. AGS is a well-established representative model of intestinal-type gastric adenocarcinoma, corresponding to the CIN molecular subtype, the most prevalent subtype, accounting for approximately 50% of all STAD cases. Importantly, our TCGA/UALCAN analysis revealed broad upregulation of SHP2 and multiple RTKs in the STAD cohort, a finding most consistent with CIN-subtype predominance within the dataset, given that CIN tumours are characterised by marked RTK amplification and constitutive RAS/MAPK/PI3K-AKT pathway activation, rendering this subtype the most amenable to SHP2-targeted therapeutic intervention. The AGS cell line therefore represents a clinically and molecularly relevant model for the initial mechanistic evaluation of NSC 57774 as a SHP2 inhibitor in gastric cancer. Future studies should nonetheless extend these findings to additional gastric cancer cell lines representing diverse molecular subtypes, including MKN45, as well as three-dimensional spheroid models, patient-derived organoids, and ultimately in vivo xenograft models, to comprehensively establish the therapeutic applicability of NSC 57774 across the full molecular spectrum of gastric carcinoma.

Beyond the single cell line limitation discussed above, the present study does not include direct SHP2 phosphatase activity assays or genetic SHP2 ablation experiments such as siRNA-mediated knockdown or CRISPR-based knockout. However, it is important to emphasize that the direct biochemical validation of NSC 57774 as a SHP2 inhibitor was rigorously established in our previously published work through fluorometric phosphatase activity assays and structure-based molecular docking against the SHP2 catalytic domain [14] rendering its repetition in the current study unnecessary. The present work was therefore deliberately designed to extend this validated inhibitory activity into the gastric cancer context by focusing on the downstream oncogenic signaling consequences of SHP2 inhibition, an aspect that had not been previously investigated for this compound. Future studies will nonetheless incorporate SHP2 siRNA knockdown, genetic rescue experiments, and multi-cell-line validation to fully consolidate the specificity and broader applicability of these findings.

A further limitation pertains to the selection of the Human dermal fibroblasts (HDF), a non-cancerous cell line, to evaluate the differential cytotoxicity of NSC 57774. While primary gastric mucosal epithelial cells would represent the most physiologically appropriate comparator, their use was not feasible due to the well-recognised limitations of primary gastric epithelial cell culture systems, which to date lack robust and reproducible in vitro maintenance protocols that adequately preserve their differentiated phenotype over extended experimental periods. HDF were therefore employed as a widely accepted non-malignant surrogate for cytotoxicity assessment and selectivity index determination, consistent with established practice in the anticancer compound evaluation literature [14]. Importantly, the consistently higher IC50 values of NSC 57774 in HDF compared to AGS cells across all time points demonstrate a meaningful selectivity index, providing preliminary but compelling evidence that NSC 57774 preferentially targets malignant over non-malignant cells. Future studies will incorporate normal gastric epithelial cell lines or patient-derived gastric organoids as more physiologically relevant and anatomically matched comparators to more rigorously establish the therapeutic window of NSC 57774 in a gastric-specific cellular context.

Finally, and perhaps most importantly from a translational perspective, the present study evaluated NSC 57774 exclusively as a single-agent therapy in a simplified monoculture system, without exploring its potential within combination therapeutic strategies or its interactions with the tumor immune microenvironment. This represents a significant limitation given the emerging clinical and preclinical evidence supporting the combination of SHP2 inhibitors with immune checkpoint blockade particularly anti-PD-1/PD-L1 therapy as a highly promising strategy in solid tumors [25]. SHP2 is known to play a critical role in modulating T-cell activation and restraining anti-tumor immune responses through dephosphorylation of key immune signaling intermediates within the PD-1/PD-L1 axis, suggesting that its inhibition could synergistically potentiate the efficacy of immune checkpoint inhibitors beyond its direct anti-tumor effects on cancer cells. Furthermore, monotherapy with SHP2 inhibitors may be insufficient to completely and durably abrogate RTK-MAPK pathway activation due to adaptive feedback resistance mechanisms, further underscoring the rationale for rational combination approaches. Collectively, the limitations outlined above define a clear and ambitious roadmap for the future development of NSC 57774: from multi-cell-line and in vivo validation, through genetic specificity confirmation, to the ultimate evaluation of its potential as a component of combination immunotherapy regimens for SHP2-driven gastric carcinoma.

## Conclusion

In this proof-of-concept in vitro study, our findings demonstrate that NSC 57774 is a potent SHP2 inhibitor with significant anti-tumor activity in the AGS intestinal-type gastric adenocarcinoma cell line, a well-established representative model of the CIN molecular subtype. Our study establishes NSC 57774 as a promising next-generation SHP2 inhibitor with substantial therapeutic potential in gastric cancer. NSC 57774 exerts broad modulatory effects on pivotal signaling pathways governing apoptosis, cell survival, migration and inflammation, notably impairing ERK, AKT and STAT3 cascades that underlie tumor progression and metastasis. The inhibitor also potently suppresses NF-κB and phospho-p38 activity while promoting cleaved caspase-3 expression, collectively underscoring its pro-apoptotic and anti-inflammatory efficacy. Notably, NSC 57774 demonstrated superior anti-tumor activity compared to both the established SHP2 inhibitor NSC 87877 and the chemotherapeutic agent doxorubicin, significantly reducing cell viability and migration in gastric cancer cells. While these findings provide a compelling mechanistic rationale for NSC 57774 as a SHP2-targeted agent in gastric cancer, we acknowledge that the restriction to a single cell line represents a scientific gap that must be addressed before broader therapeutic conclusions can be drawn. Future research should prioritize validation in additional molecularly distinct gastric cancer cell lines, alongside three-dimensional spheroid models, patient-derived organoids, and in vivo xenograft systems, to elucidate its molecular mechanisms of action, evaluating in vivo efficacy and investigating its utility within combination regimens to overcome resistance and enhance clinical outcomes across the full molecular spectrum of gastric carcinoma.

## Supporting information

S1 Fig Dose-dependent cytotoxic effect of NSC 87877 on AGS gastric cancer cells over 24, 48 and 72 hours as determined by MTT assay. IC₅₀ values calculated at 24, 48 and 72 hours.
(PDF)

S2 Fig Dose-dependent cytotoxic effect of NSC 57774 on Human dermal fibroblasts (HDF) over 24, 48 and 72 hours as determined by MTT assay. IC₅₀ values calculated at 24, 48 and 72 hours.
(PDF)

S1 File Raw images. (PDF)
