# Supplementary material for: Mechanistic evaluation of NSC 57774 as a SHP2 inhibitor in gastric cancer: Multi-pathway signaling modulation in vitro
Source: PLoS One. 2026 Jul 30;21(7):e0354605. doi: 10.1371/journal.pone.0354605 (PMC13422832; doi:10.1371/journal.pone.0354605)
Supplement: S1 Fig — IC₅₀ values calculated at 24, 48 and 72 hours. (PDF) [file pone.0354605.s001.pdf]

NSC 87877 (24 h)

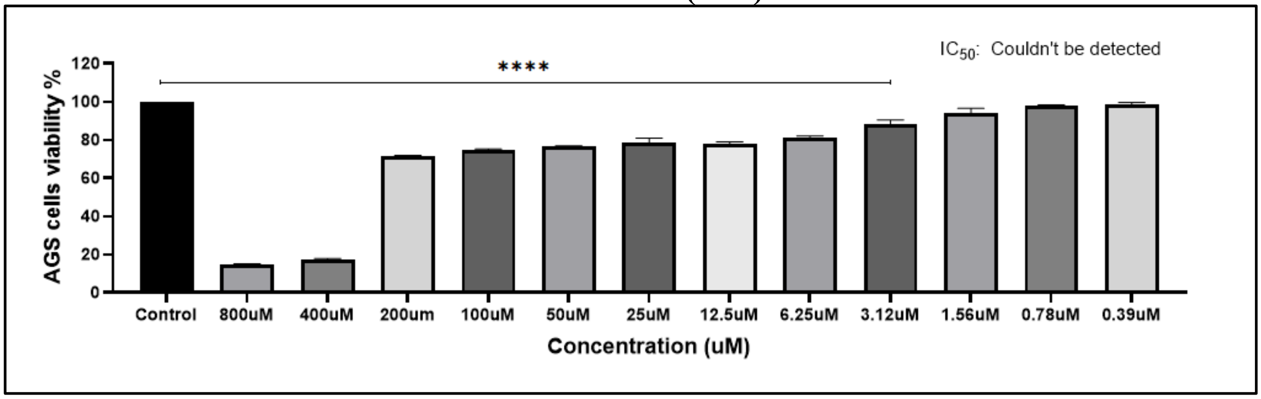

NSC 87877 (48 h)

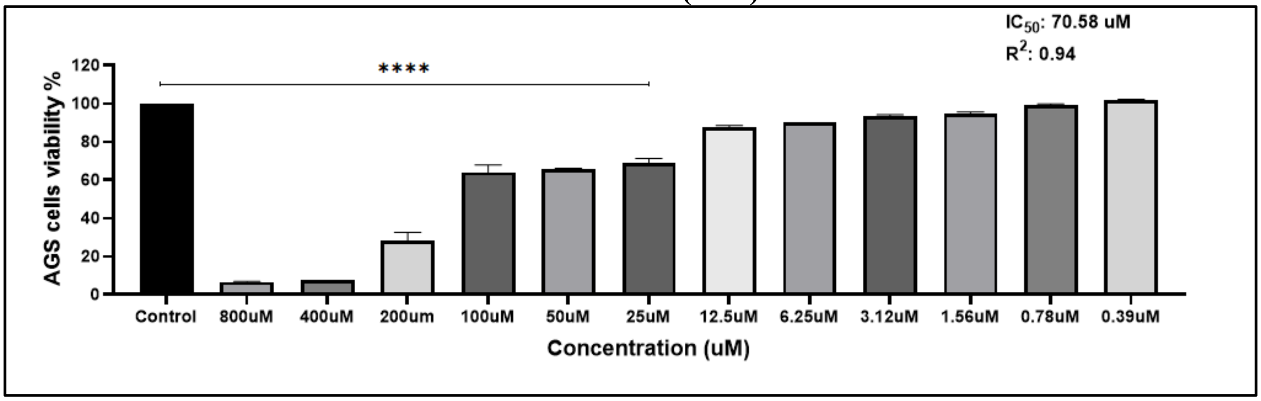

NSC 87877 (72 h)

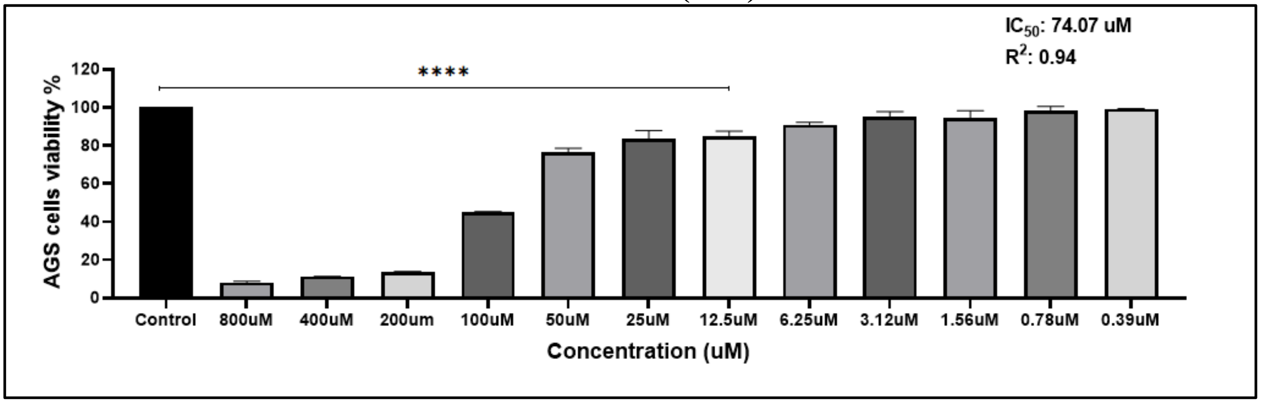

S1 Fig: Dose-dependent cytotoxic effect of NSC 87877 on AGS gastric cancer cells over 24, 48 and 72 hours as determined by MTT assay. IC_{50} values calculated at 24, 48 and 72 hours.
