# Supplementary material for: Mechanistic evaluation of NSC 57774 as a SHP2 inhibitor in gastric cancer: Multi-pathway signaling modulation in vitro
Source: PLoS One. 2026 Jul 30;21(7):e0354605. doi: 10.1371/journal.pone.0354605 (PMC13422832; doi:10.1371/journal.pone.0354605)
Supplement: S1 File — (PDF) [file pone.0354605.s003.pdf]

# Supplemental Raw Blot Images

- **Supplemental Raw Blot Images B1**

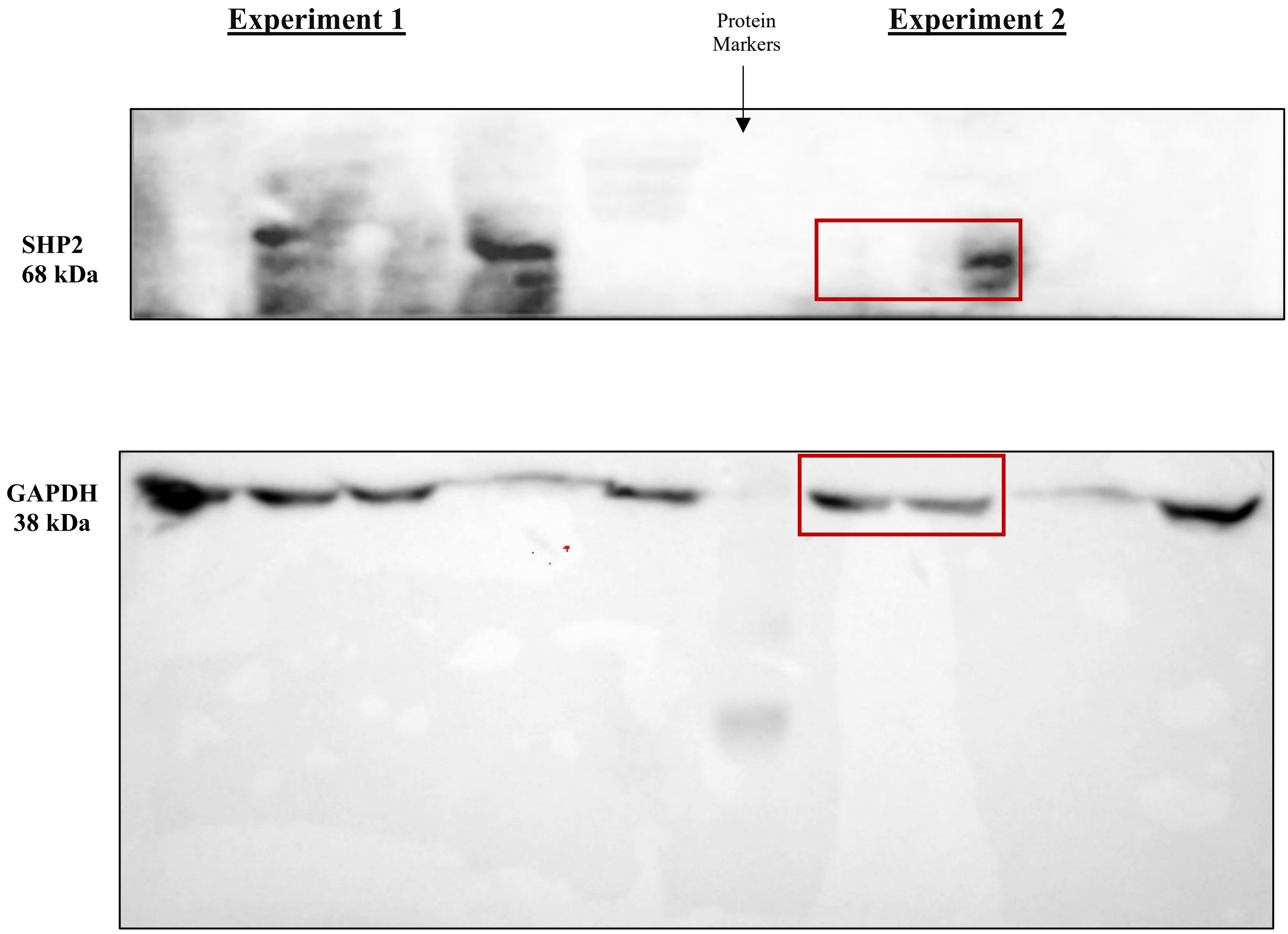

- **Supplemental Raw Blot Images B2**

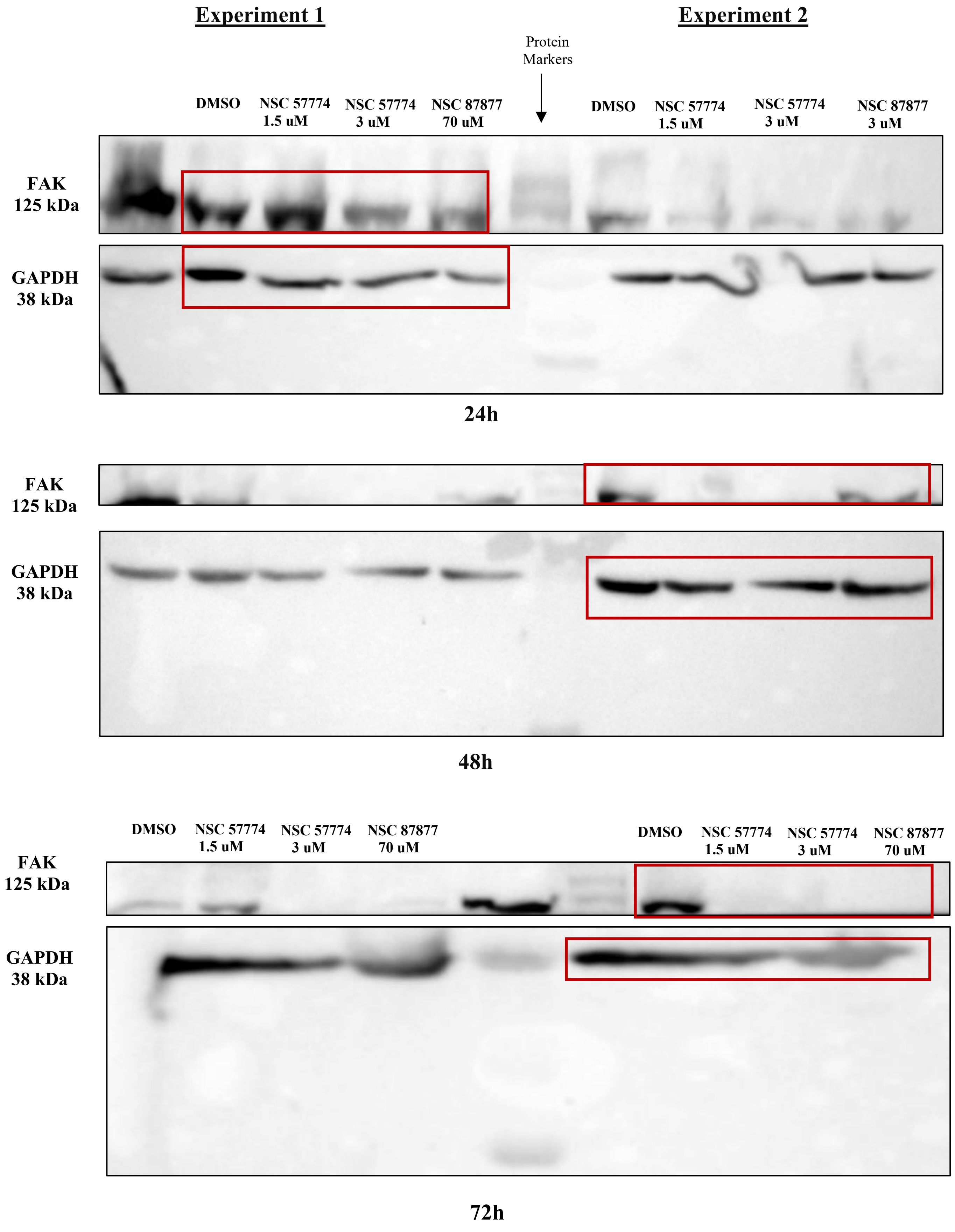

- **Supplemental Raw Blot Images B3**

**<u>Experiment 1</u>** **24h** **<u>Experiment 2</u>**

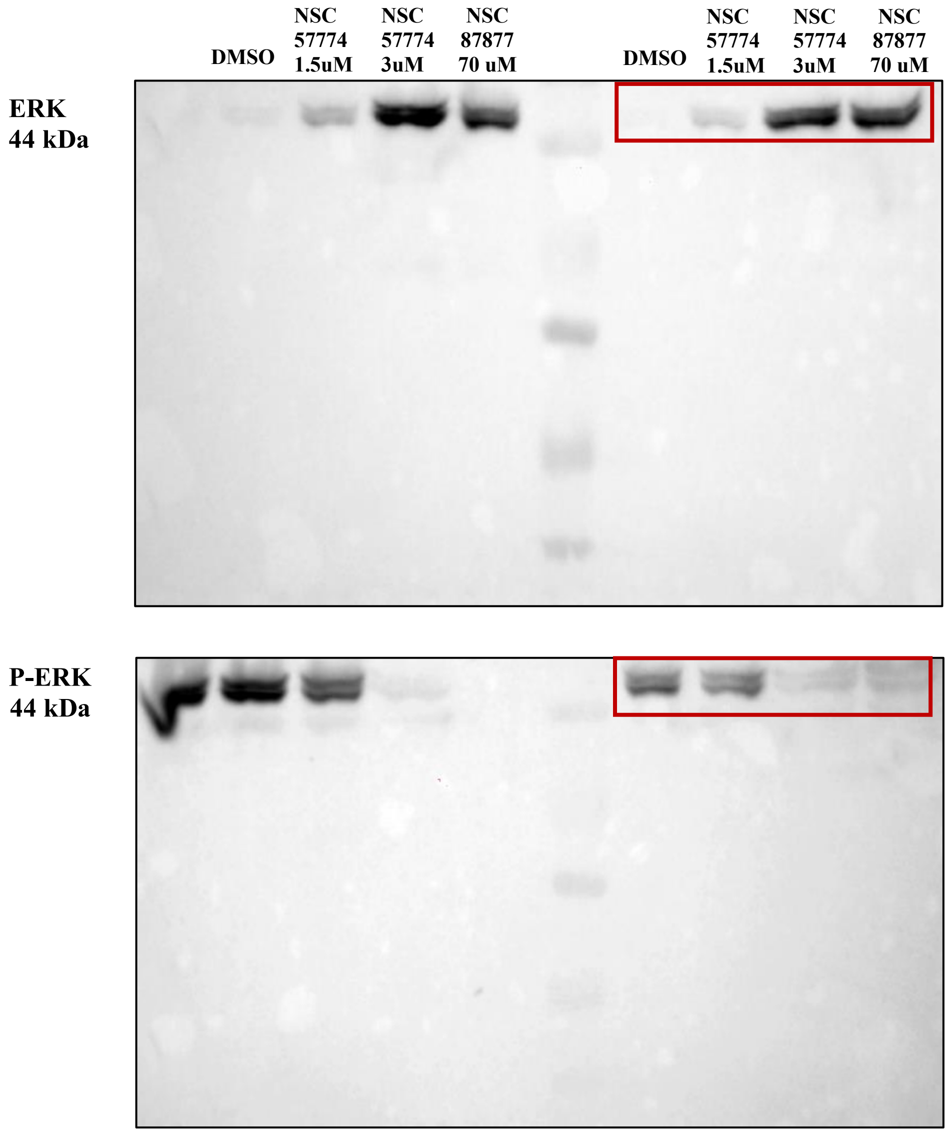

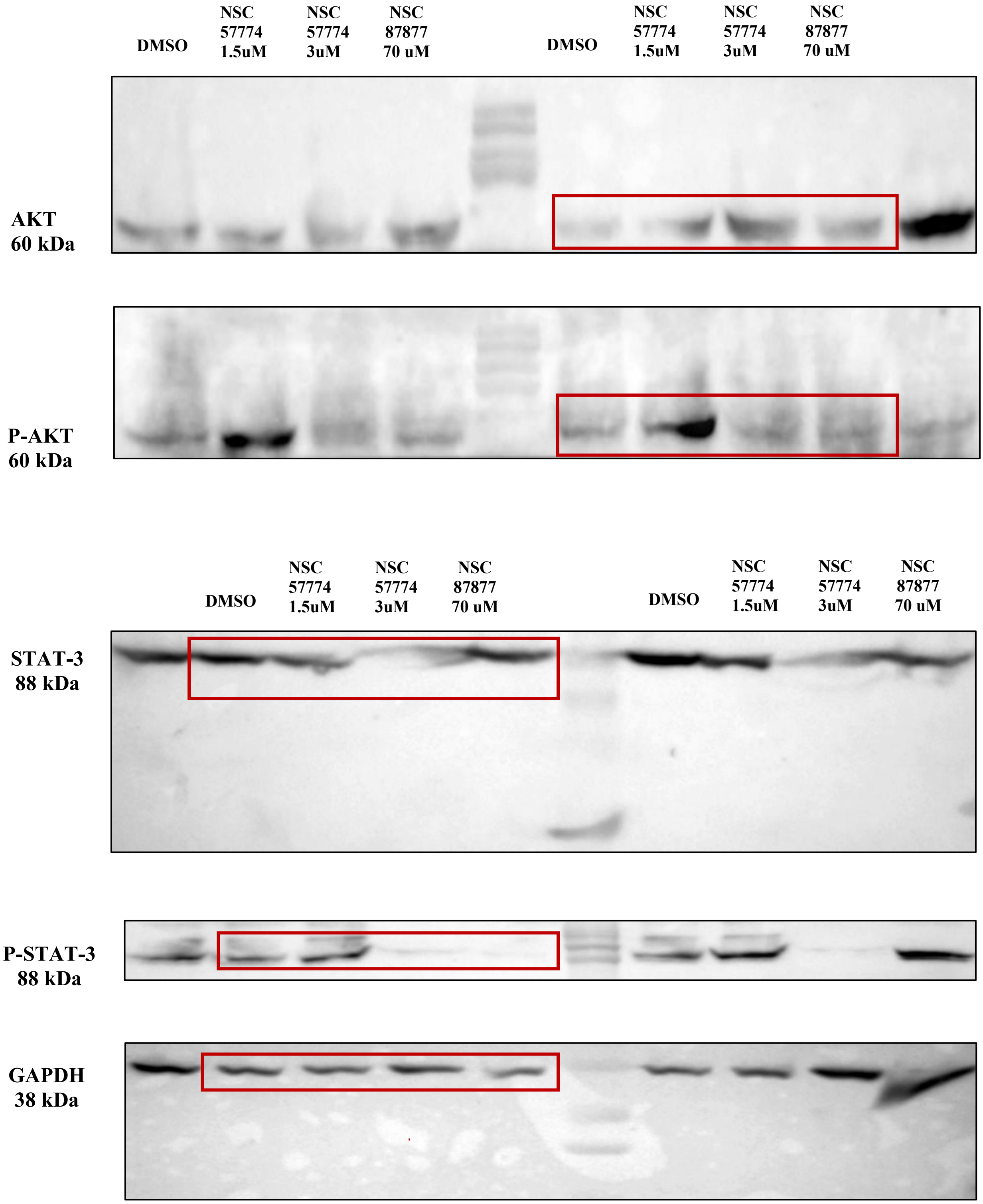
DMSO
NSC 57774 1.5uM
NSC 57774 3uM
NSC 87877 70 uM
DMSO
NSC 57774 1.5uM
NSC 57774 3uM
NSC 87877 70 uM
AKT 60 kDa
P-AKT 60 kDa
DMSO
NSC 57774 1.5uM
NSC 57774 3uM
NSC 87877 70 uM
DMSO
NSC 57774 1.5uM
NSC 57774 3uM
NSC 87877 70 uM
STAT-3 88 kDa
P-STAT-3 88 kDa
GAPDH 38 kDa

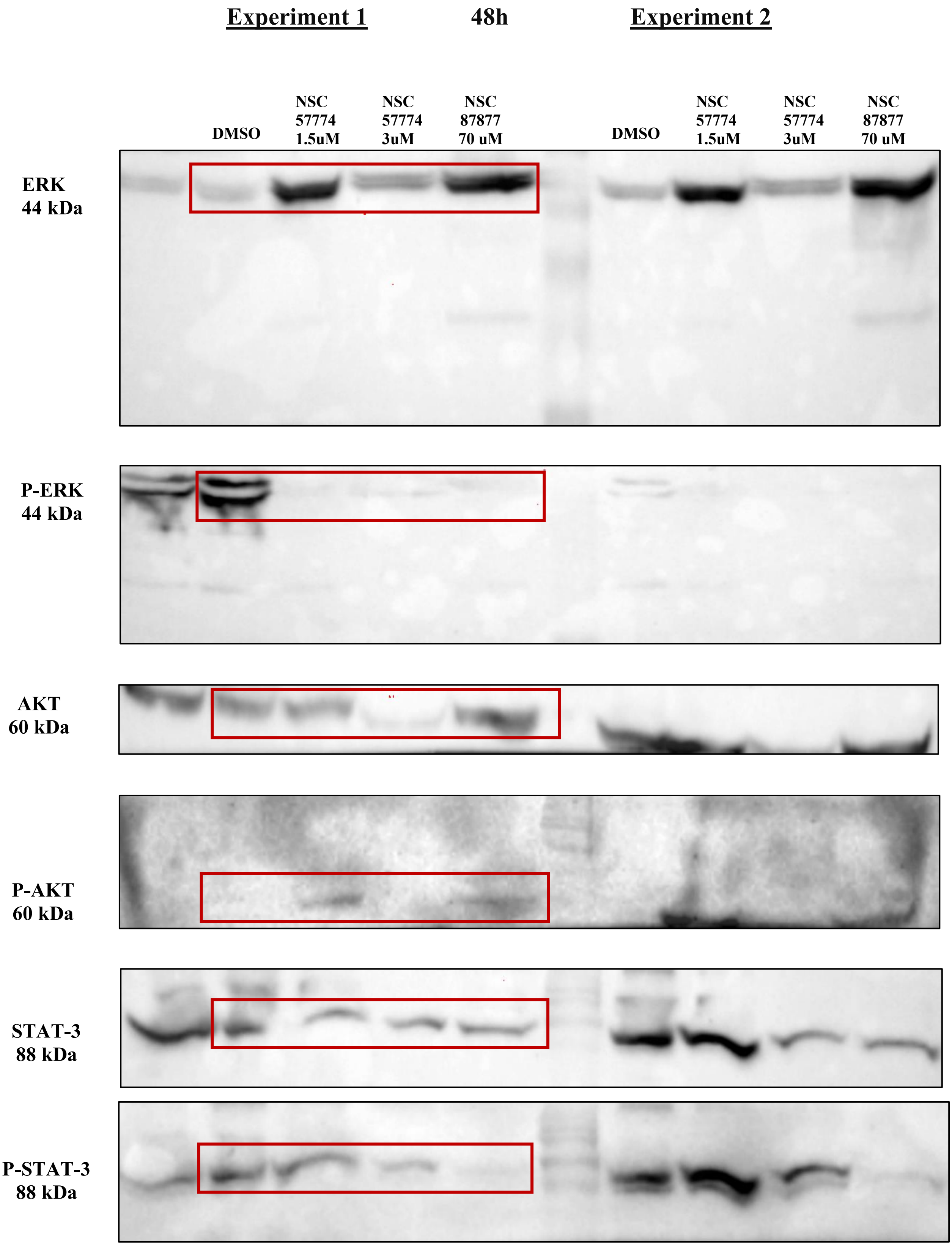
Experiment 1
48h
Experiment 2
DMSO
NSC 57774 1.5uM
NSC 57774 3uM
NSC 87877 70 uM
DMSO
NSC 57774 1.5uM
NSC 57774 3uM
NSC 87877 70 uM
ERK 44 kDa
P-ERK 44 kDa
AKT 60 kDa
P-AKT 60 kDa
STAT-3 88 kDa
P-STAT-3 88 kDa

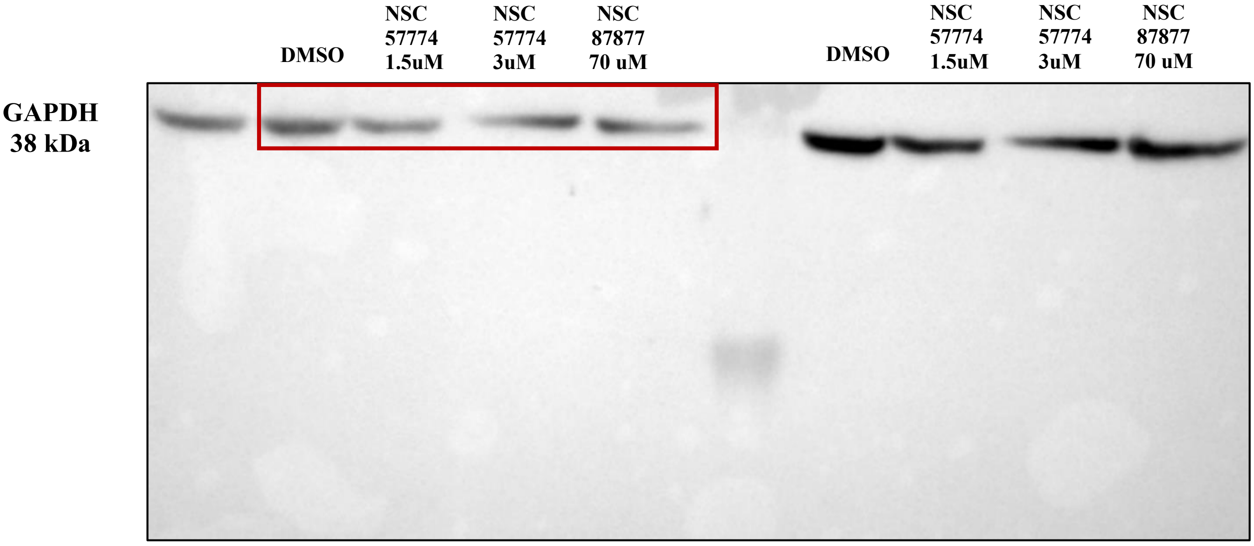
DMSO
NSC 57774 1.5uM
NSC 57774 3uM
NSC 87877 70 uM
DMSO
NSC 57774 1.5uM
NSC 57774 3uM
NSC 87877 70 uM
GAPDH
38 kDa

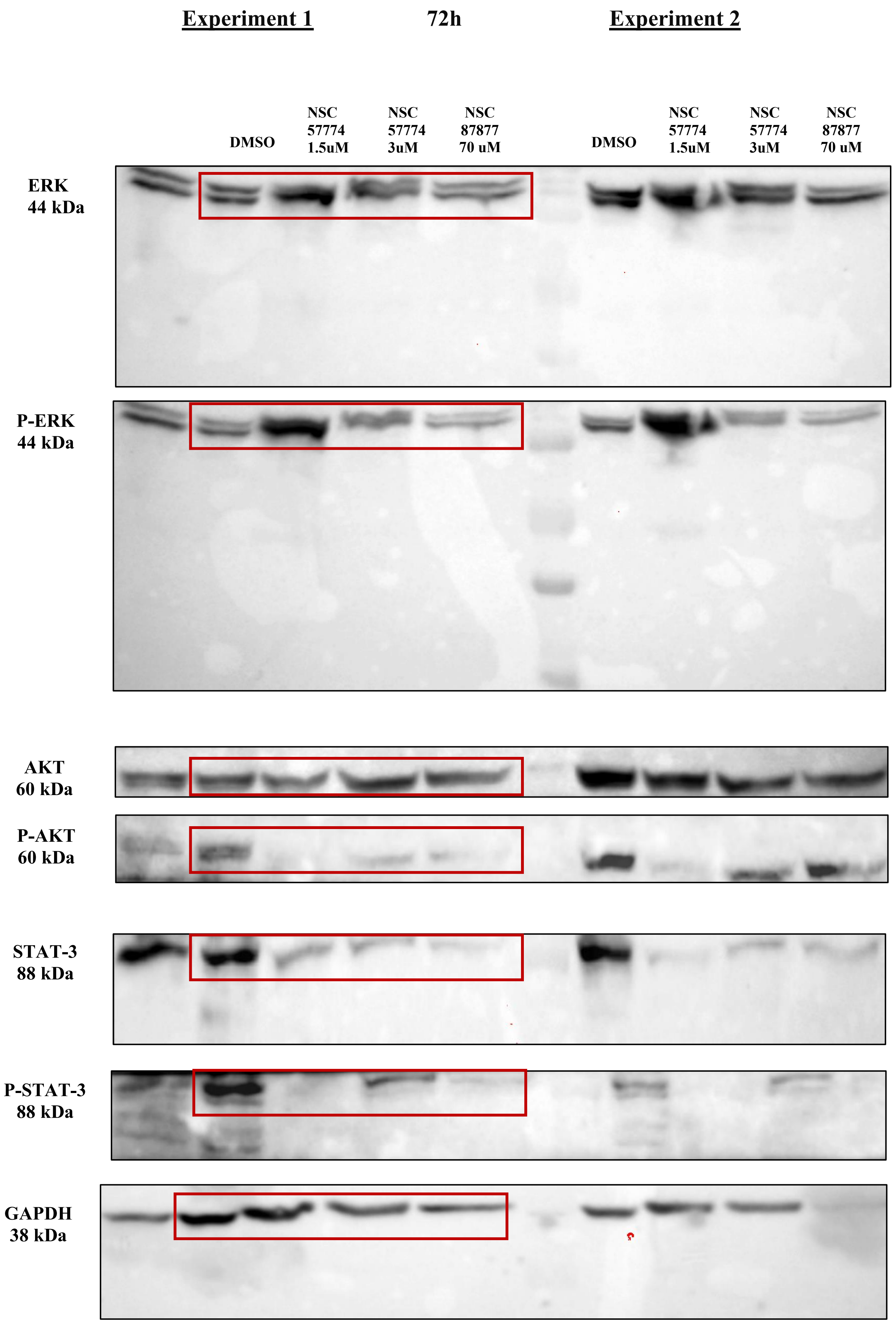
Experiment 1
72h
Experiment 2
DMSO
NSC 57774 1.5uM
NSC 57774 3uM
NSC 87877 70 uM
DMSO
NSC 57774 1.5uM
NSC 57774 3uM
NSC 87877 70 uM
ERK 44 kDa
P-ERK 44 kDa
AKT 60 kDa
P-AKT 60 kDa
STAT-3 88 kDa
P-STAT-3 88 kDa
GAPDH 38 kDa

- **Supplemental Raw Blot Images B4**

**Experiment 1** **24h** **Experiment 2**

| DMSO | NSC 57774 1.5uM | NSC 57774 3uM | NSC 87877 70 uM | DMSO | NSC 57774 1.5uM | NSC 57774 3uM | NSC 87877 70 uM |
|---|---|---|---|---|---|---|---|

**Cas 3**
**19 kDa**

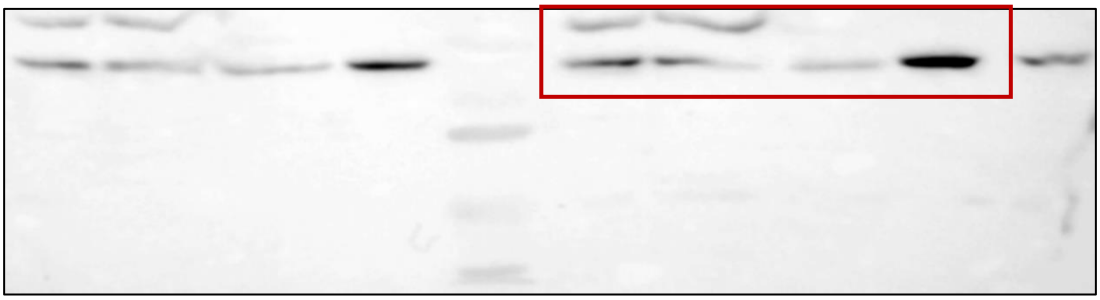

| DMSO | NSC 57774 1.5uM | NSC 57774 3uM | NSC 87877 70 uM | DMSO | NSC 57774 1.5uM | NSC 57774 3uM | NSC 87877 70 uM |
|---|---|---|---|---|---|---|---|

**NF-κB**
**65 kDa**

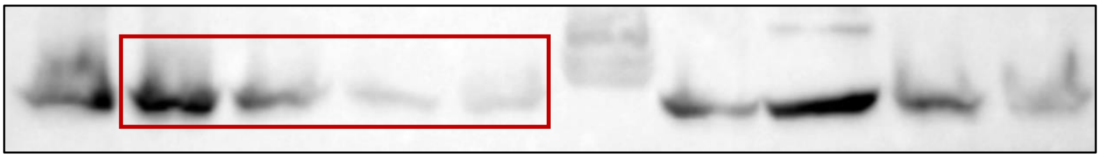

**p38**
**38 kDa**

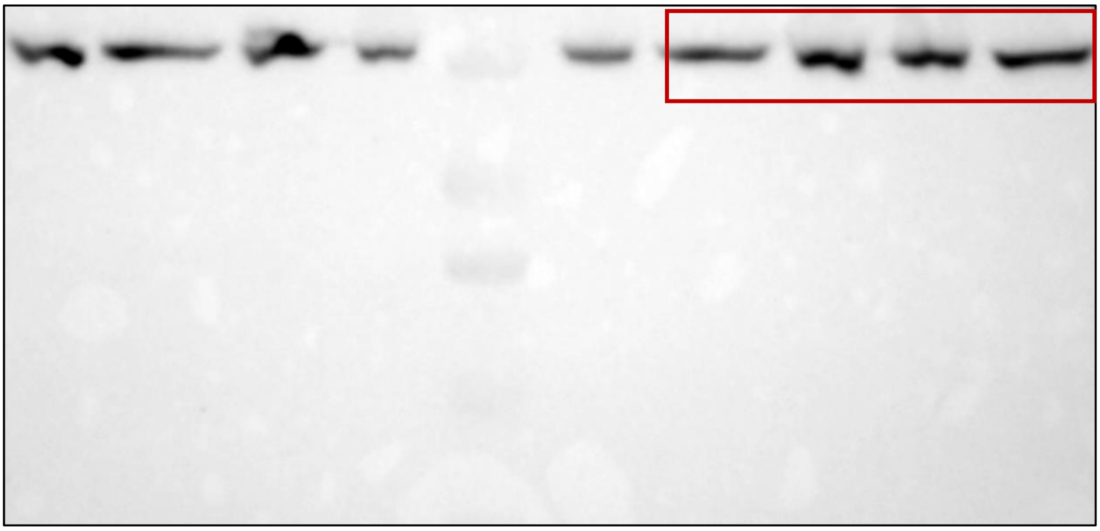

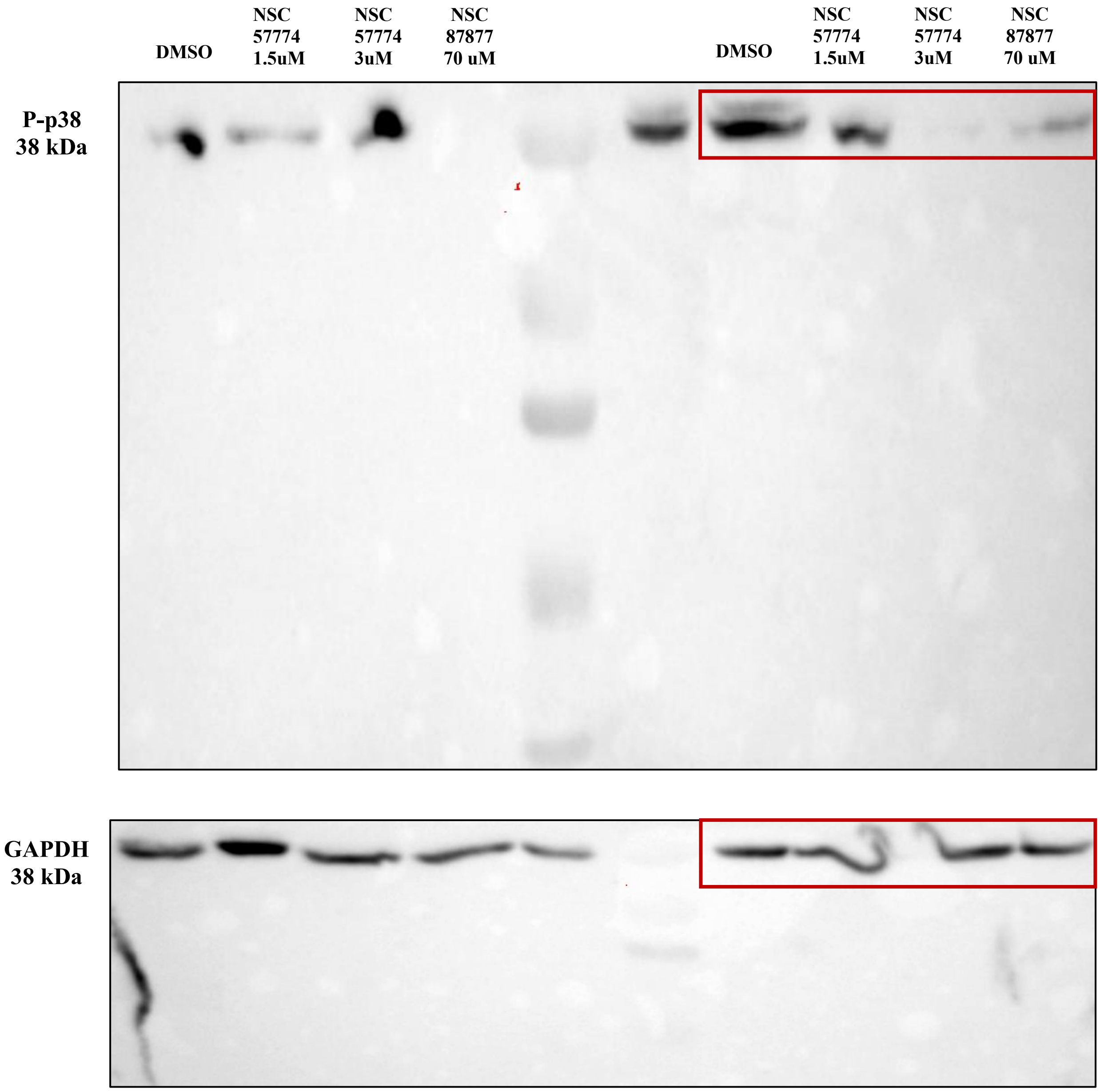
DMSO
NSC 57774 1.5uM
NSC 57774 3uM
NSC 87877 70 uM
DMSO
NSC 57774 1.5uM
NSC 57774 3uM
NSC 87877 70 uM
P-p38 38 kDa
GAPDH 38 kDa

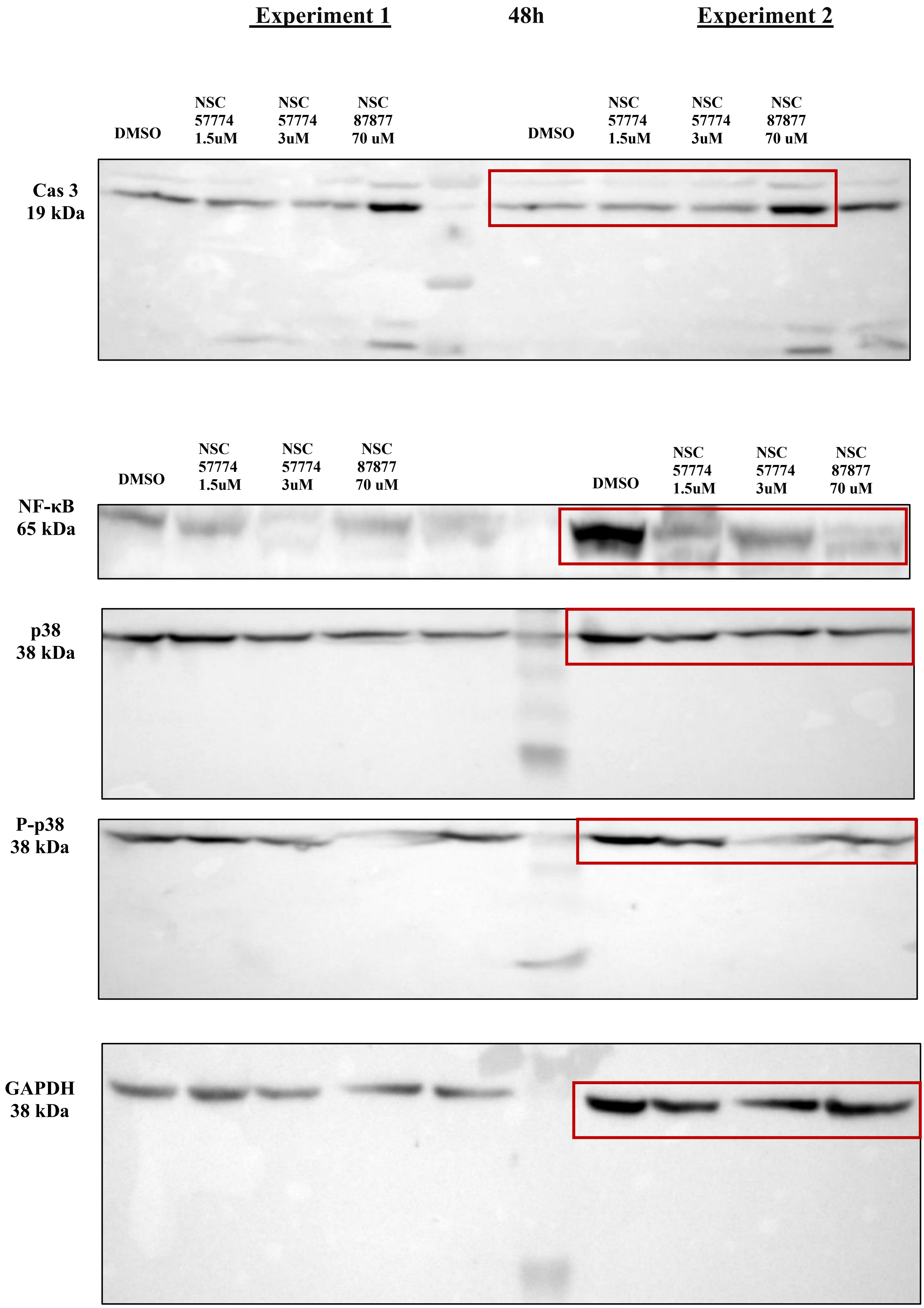
Experiment 1
48h
Experiment 2
DMSO
NSC 57774 1.5uM
NSC 57774 3uM
NSC 87877 70 uM
DMSO
NSC 57774 1.5uM
NSC 57774 3uM
NSC 87877 70 uM
Cas 3 19 kDa
DMSO
NSC 57774 1.5uM
NSC 57774 3uM
NSC 87877 70 uM
DMSO
NSC 57774 1.5uM
NSC 57774 3uM
NSC 87877 70 uM
NF-κB 65 kDa
p38 38 kDa
P-p38 38 kDa
GAPDH 38 kDa

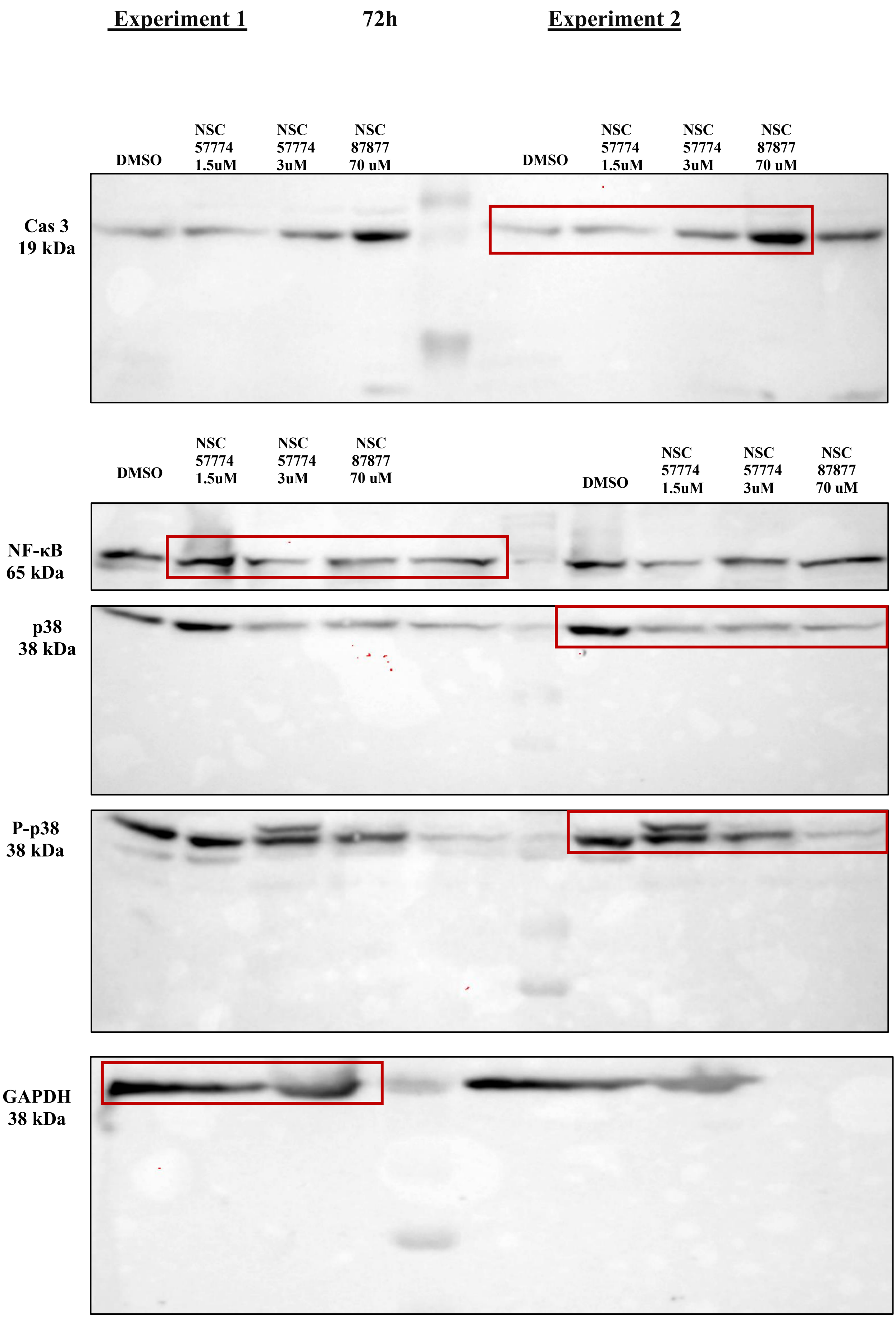
Experiment 1
72h
Experiment 2
DMSO
NSC 57774 1.5uM
NSC 57774 3uM
NSC 87877 70 uM
DMSO
NSC 57774 1.5uM
NSC 57774 3uM
NSC 87877 70 uM
Cas 3 19 kDa
DMSO
NSC 57774 1.5uM
NSC 57774 3uM
NSC 87877 70 uM
DMSO
NSC 57774 1.5uM
NSC 57774 3uM
NSC 87877 70 uM
NF-κB 65 kDa
p38 38 kDa
P-p38 38 kDa
GAPDH 38 kDa
